# Comparison of two small-strain concepts: ISA and intergranular strain applied to barodesy

**DOI:** 10.1007/s11440-022-01454-3

**Published:** 2022-04-06

**Authors:** Merita Tafili, Gertraud Medicus, Manuel Bode, Wolfgang Fellin

**Affiliations:** 1 Ruhr-University Bochum, Chair of Soil Mechanics, Foundation Engineering and Environmental Geotechnics, Universitätsstr. 150, 44801 Bochum, Germany; 2 University of Innsbruck, Unit of Geotechnical Engineering, Technikerstr. 13a, 6020 Innsbruck, Austria

**Keywords:** Barodesy, Cyclic loading, ISA, Intergranular strain, Small-strain stiffness

## Abstract

The intergranular strain concept (IGS) and intergranular strain anisotropy formulation (ISA) are state of the art extensions to describe small-strain effects. The main conceptional difference between ISA and IGS is the purely elastic strain range introduced by ISA. In addition, the ISA formulation used in this article includes an additional state variable in order to reduce accumulation effects for cyclic loading with a larger number of repetitive cycles. Barodesy is enhanced here with ISA to improve its small-strain predictions. The performance of this new model is compared with barodesy enhanced with IGS. It turned out that the small-strain extensions do not negatively influence predictions under monotonic loading. Differences between ISA and ISG are only remarkable for very small-strain cycles and even there they are negligible for certain parameter values.

**Supplementary Information:**

The online version contains supplementary material available at 10.1007/s11440-022-01454-3.

## Introduction

It is essential to understand and constitutively describe the mechanical behaviour of fine-grained soils under cyclic loading for many applications such as offshore wind turbines subjected to wave cycles and wind cycles, geotechnical structures subjected to repetitive traffic and high-speed train loads, or even earthquake loading.

In finite element simulations, it is important to use a constitutive model that includes stress–strain relations that are appropriate for the given problem. In order to accurately address the small-strain stiffness and reproduce the soil behaviour under cyclic loading [36] using hypoplastic models, Niemunis & Herle in 1997 [24, 26] proposed the concept of the intergranular strain (IGS). This extension aimed to remedy the hypoplastic saw-tooth-like paths (ratcheting), which yields an excessive accumulation of deformation predicted for small stress cycles. Even though the IGS approach considerably reduces ratcheting, it does not vanish completely. Furthermore, the strain amplitude at cyclic mobility during undrained cyclic shearing grows too slow. [17] formulated the hypoplastic model with IGS for sand under the framework of anisotropic critical state theory (ACST) introducing an evolving fabric tensor as a remedy for these shortcomings when dealing with granular materials. In this course, [39] addressed the description of inherent anisotropy of granular soils in hypoplastic models using the ACST. [14] included strength anisotropy into the hypoplastic model [19] by rotating the asymptotic state boundary surface.

Fuentes and Triantafyllidis [7] reformulated the intergranular strain concept in 2015 and named it ISA-model, often denoted also as ISA-plasticity. This new approach introduces an elastoplastic intergranular strain, and hence, it includes a yield surface in the intergranular strain space. It was first introduced for granular materials using projections in the stress space for the mechanical model. Table 1 provides a summary of the models developed under the ISA-plasticity framework. The second version for sands [27] was determined by the coupling between the ISA concept of the intergranular strain and the well-known hypoplastic model for granular materials developed by von Wolffersdorff [38]. The latter one loses the ability to account for cyclic mobility effects, and hence, liquefaction phenomena are not reproduced. In 2019 [10], ISA has been extended by introducing an additional state variable that permits the detection of cyclic mobility paths.

For fine-grained soils, a first model has been developed in 2015 [8] and extended in 2017 [9, 29] by introducing a time and rate dependency according to [33, 34]. In both models for fine-grained soils, the intergranular strain formulation of sand [27] is used. Tafili and Triantafyllidis [28, 31] enhanced the ISA-plasticity to capture the specific behaviour of soft soils and introduced also the inherent anisotropy effects [30, 32] into the mechanical model formulation (AVISA model). In 2020, the ISA-plasticity has been coupled with the hypoplastic model for fine-grained soils developed by Mašín [11]. This model also accounts for the inherent anisotropy of fine-grained soils, but does not introduce rate- and time-dependent effects.

**Table 1:** Summary of the models developed under the ISA-plasticity framework

Name	Material	Comment	Literature
ISA—Sand (2015)	Sand	Uses projections in the stress space	[7]
ISA—HP Clay (2015)	Clay	Time and rate independent	[8]
ISA—HP Wolffersdorff (2016)	Sand	Without projections in the stress space	[27]
ISA—visco HP Clay (2017)	Clay	Inclusion of rate and time dependency	[9]
ISA—HP Wolffersdorff (2019)	Sand	Inclusion of cyclic mobility effects	[10]
AVISA—aniso visco HP Clay (2020)	Clay	Inclusion of inherent anisotropy, rate- and time-dependent	[31]
ISA—HP Clay (2020)	Clay	Inclusion of inherent anisotropy	[11]

Barodesy is a constitutive model, which is attractive for engineering purposes due to the reduced number of parameters and also the simplicity of their determination. A fundamental drawback of the model is, however, the inability of describing the behaviour of soils under cyclic loading. Therefore, in this article, barodesy for clay [22] is extended with ISA according to [27], hereafter denoted as ISA-B. Comparison is made with the IGS [26] extension for barodesy [1], hereafter denoted as IGS-B. We decided to use the most common versions of ISA [27] and IGS [26] to investigate conceptual differences. The main difference between ISA and IGS is that only ISA includes a purely elastic strain range. In addition, the ISA version used in this article includes an additional state variable in order to reduce accumulation effects under cyclic loading with a large number of repetitive cycles.

In this article, the similarities and differences between ISA-B and IGS-B are discussed. In addition, the models are compared with (cyclic) experiments of Kaolin and Lower Rhine clay.

## Barodesy, IGS and ISA

### Barodesy

The constitutive model barodesy differs fundamentally from conventional elastoplastic models: it does not distinguish between elastic and plastic deformations and does not require expressions such as a yield function, a plastic potential or a flow rule. The stress rate is formulated as a tensorial function of the current stress, stretching and the void ratio, i.e. ${\mathring{{\mathbf {T}}}}={\mathbf {h}}({\mathbf {T}}, {\mathbf {D}}, e)$. Thus, barodesy has certain similarities with hypoplasticity [19, 38].

Basic concepts from Critical State Soil Mechanics are included in the mathematical formulation of barodesy [22]:

(i) Critical stress states of barodesy virtually coincide with the Matsuoka–Nakai failure criterion [6].
(ii) A stress-dependent critical void ratio $e_c$ (CSL according to [18]) in the $p'$-*e* plot enables to distinguish between normally to slightly overconsolidated soil ($e>e_c$) and highly overconsolidated soil ($e<e_c$).
(iii) The isotropic normal compression line (NCL) according to [4] defines normally consolidated states.

Further details of the mathematical formulation of barodesy are given in [15, 16, 21–23]. For the calibration procedure, we refer to [22]. “Appendix B” summarizes all equations of barodesy for clay [22].

### Intergranular strain concept (IGS)

The intergranular strain concept (IGS) was introduced by Niemunis and Herle [26] to improve the description of soil behaviour under cyclic loading with hypoplastic models and to capture the effects of small-strain stiffness. The short-term deformation history is stored as an additional tensorial state variable, the so-called intergranular strain ${\mathbf {h}}$. After a change in the deformation direction, the stiffness of the hypoelastic (*linear*) part of the hypoplastic model is used, however, increased by a scalar factor. For a constant stretching after the change in the deformation direction, the IGS performs a reduction of the increased stiffness and an interpolation between the linear part and the full hypoplastic model. Because of its mathematical formulation, the original IGS by Niemunis and Herle [26] is not directly applicable to non-hypoplastic models. To overcome this, Bode et al. [1] proposed a formulation of the IGS to allow for an application to barodesy. Details of this formulation and also of the general IGS formulation can be found in [1, 2, 24, 26, e.g.].

### ISA-plasticity for the small-strain stiffness

In the following lines, the ISA-plasticity formulation proposed by Poblete et al. [27] is briefly summarized using the notation depicted in Sect. A. Some explanations about the state variables and parameters responsible for the reproduction of cumulative soil behaviour are provided. For further details, we refer to the original works published in [7, 9, 27].

**Fig. 1:**
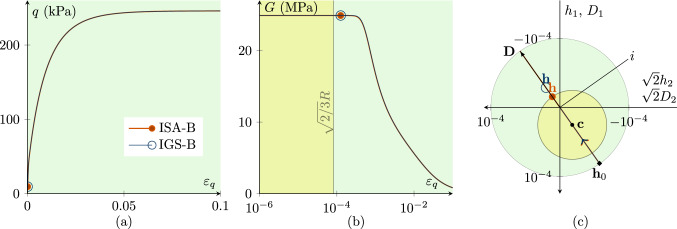
CU test with $${\mathbf {h}}_0=\sqrt{2/3}R \begin{pmatrix} 1 &{} 0 &{} 0 \\ 0 &{} -1/2 &{} 0 \\ 0 &{} 0 &{} -1/2 \\ \end{pmatrix}\,\text {and}\, {\mathbf {c}}_0={\mathbf {h}}_0/2$$ with fully mobilized intergranular strain $||{\mathbf {h}}||=R$, performing a $180^\circ$ strain path reversal; $\chi _0=\chi =\chi _{\rm max}$ is set to 20; $p'_0=200$ kPa; $\text{ OCR}_0=2.5$. The simulations with IGS-B and ISA-B almost coincide. **a** Deviatoric stress *q*–deviatoric strain $\varepsilon _q$ plot, **b** the secant shear stiffness *G*–deviatoric strain $\varepsilon _q$ plot, **c** Rendulic planes of intergranular strain ${\mathbf {h}}$ and stretching ${\mathbf {D}}$ for the state indicated by circles in (**a, b**). The line *i* refers to the hydrostatic direction of ${\mathbf {D}}$ and ${\mathbf {h}}$ (see Supplementary file 1: Animation related to Figure 1)

The main feature of the ISA-plasticity formulation is the incorporation of the elastic locus, see Fig. 1c, as a strain-type yield surface. It describes a hypersphere with diameter *R* as a material parameter and depends on the intergranular strain tensor ${\mathbf {h}}$ and the kinematic hardening tensor ${\mathbf {c}}$

1 $$\begin{aligned} F_H=\Vert {\mathbf {h}}-{\mathbf {c}}\Vert -R/2=0 \end{aligned}$$

The evolution of the intergranular strain tensor is described in accordance with elastoplastic formulations introducing the consistency parameter ${\dot{\lambda }}_H$ for $\dot{F}_H=0$ and the associated flow direction ${\mathbf {N}}=\partial F_H/\partial {\mathbf {h}}$

2 $$\begin{aligned} \dot{{\mathbf {h}}}={\mathbf {D}}-{\dot{\lambda }}_H{\mathbf {N}}. \end{aligned}$$

Once the yield surface is reached, its centre evolves towards the bounding surface (BS)

3 $$\begin{aligned} F_{Hb}=\Vert {\mathbf {h}}\Vert - R=0 \end{aligned}$$

governed by the kinematic hardening state variable ${\mathbf {c}}$ and its evolution equation

4 $$\begin{aligned} {\dot{{\mathbf {c}}}}={\dot{\lambda }}_H\,\beta \,(R\,{{\mathbf {D}}}^0-{\mathbf {c}})/R \end{aligned}$$

whereby $\beta$ is a material parameter controlling the hardening rate.

According to the ISA-plasticity, the BS is reached after applying stretching in a constant direction ${{\mathbf {D}}}^0$. In order to reduce the plastic strains between this “fully mobilized” state and the elastic condition (e.g. due to an unloading process), the scalar function $y_h$ has been introduced

5 $$\begin{aligned}&0\le y_h=\rho ^{\chi }\langle {\mathbf {N}}:{{\mathbf {D}}}^0\rangle \le 1, \nonumber \\&\quad \rho =1-\dfrac{\Vert {\mathbf {h}}_b-{\mathbf {h}}\Vert }{ R}, \end{aligned}$$

whereby ${\mathbf {h}}_b=R\,{\mathbf {N}}$ is the image tensor of ${\mathbf {h}}$ at the bounding surface.

According to physical experiments, e.g. [28, 35, 37], the cumulative behaviour of the soil reduces for increasing number of consecutive cycles before reaching the critical state. To describe this effect, Poblete et al. [27] introduced the so-called internal variable for cyclic history $0\le \varepsilon _a\le 1$ to the former ISA-model [7], which indicates whether the soil has been objected to a few or several consecutive cycles. For reconstituted samples, this variable can be initialized to $\varepsilon _{a,0}=0$, because the sample has not been previously subjected to cyclic loading. As this work deals with the constitutive description of reconstituted soil, $\varepsilon _{a,0}$ was initialized to zero in all simulations. For in situ conditions, the initialization is to some extent controversial because it often cannot be established whether the soil has been previously subjected to cyclic loading. Therefore, we recommend initializing the variable to zero while simulating the entire history of previous loading. The evolution rate of the internal variable, which will then save the information about the altering of the soil subjected to consecutive cycles, is controlled through a material parameter $C_a$ and reads

6 $$\begin{aligned} {\dot{\varepsilon }}_a=C_a/R\left( 1-y_h-\varepsilon _a\right) \Vert {\mathbf {D}}\Vert . \end{aligned}$$

For a few number of cycles or for monotonic loading $y_h\rightarrow 1$, hence $\varepsilon _a$ vanishes, otherwise, for a large number of consecutive cycles $y_h\rightarrow 0\Rightarrow \varepsilon _a\rightarrow 1$.

This feature is now used to intensify the effect of the intergranular strain at larger number of cycles through the modification of the exponent $\chi$ in Eq. 5. As $y_h$ as well as the plastic accumulation rate decrease with increasing $\chi$, the relation $\chi =\chi _0+\varepsilon _a\,(\chi _{\rm max}-\chi _0)$ has been introduced in [27]. Hence, the minimum value $\chi =\chi _0$ resulting in maximal plastic strain accumulation is reached for a few number of cycles or for monotonic loading. On the other hand, a large number of cycles leads to the maximum value $\chi =\chi _{\rm max}$, which in turn reduces the plastic accumulation rate. Hence, $\chi _0$ and $\chi _{\rm max}$ are material parameters that can be calibrated for a few and many number of cycles, respectively.

To reproduce the stiffness increase due to changes in the direction of the strain path, the scalar function $m=m_R+(1-m_R)\,y_h$ is included into the ISA-plasticity, where $m_R$ is a material parameter. Note the dependence of the stiffness increase factor on $\chi$ through $y_h$.

A more recent development by [5] applied to clay hypoplasticity [19] contains a similar improvement for IGS to predict cyclic loading as introduced for ISA by [27].

### Implementation of ISA in barodesy

In this section, the ISA implementation of hypoplasticity [27] will be extended with the ideas used in the IGS implementation of barodesy [1]. The basic formulation of hypoplasticity reads

7 $$\begin{aligned} \mathring{{\mathbf {T}}}= {\boldsymbol {\mathcal {L}}}^{\rm H}\mathbin {:}{\mathbf {D}}+ {\mathbf {N}}^{\rm H} \Vert {\mathbf {D}}\Vert = \mathring{{\mathbf {T}}}_{\rm el}+ \mathring{{\mathbf {T}}}_{\rm ne}\, . \end{aligned}$$

In this formulation, the term linear in ${\mathbf {D}}$ (i.e. ${\boldsymbol {\mathcal {L}}}^{\rm H}\mathbin {:}{\mathbf {D}}$) represents an incrementally elastic response $\mathring{{\mathbf {T}}}_{\rm el}$. The other term is not linear in ${\mathbf {D}}$ and represents the non-elastic response.

The stress rate of the ISA hypoplastic model is given by

8 $$\begin{aligned} \mathring{{\mathbf {T}}}= {\boldsymbol {\mathcal {M}}}\mathbin {:}{\mathbf {D}}, \end{aligned}$$

with the material stiffness matrix

9 $$\begin{aligned} {\varvec {\mathcal {M}}} = \left\{ \begin{array}{*{20}l} m_R {\varvec {\mathcal {L}}}^{\rm H} &{} \, \text {for } F_H < 0 \text { (elastic)} \\ m \big ({\varvec {\mathcal {L}}}^{\rm H} + y_h {\mathbf {N}}^{\rm H} \otimes {\mathbf {N}}\big ) &{} \, \text {for } F_H = 0 \text { (plastic)} . \end{array} \right. \end{aligned}$$

Combining (8) and (9), using ${\mathbf {D}}= \Vert {\mathbf {D}}\Vert {\mathbf {D}}^0$ and rearranging ${\mathbf {N}}^{\rm H} \otimes {\mathbf {N}}\mathbin {:}{\mathbf {D}} = {\mathbf {N}}^{\rm H} \otimes {\mathbf {N}}\mathbin {:}\Vert {\mathbf {D}}\Vert {\mathbf {D}}^0 = ({\mathbf {N}}\mathbin {:}{\mathbf {D}}^0) {\mathbf {N}}^{\rm H} \Vert {\mathbf {D}}\Vert$ results in

10 $$\begin{aligned} \mathring{{\mathbf {T}}}= \left\{ \begin{array}{*{20}l} m_R {\boldsymbol {\mathcal {L}}}^{\rm H} \mathbin {:}{\mathbf {D}}&{} \, \text {for } F_H < 0\\ m \big ({\boldsymbol {\mathcal {L}}}^{\rm H} \mathbin {:}{\mathbf {D}}+ y_h ({\mathbf {N}}\mathbin {:}{\mathbf {D}}^0) {\mathbf {N}}^{\rm H} \Vert {\mathbf {D}}\Vert ) &{} \, \text {for } F_H = 0. \end{array} \right. \end{aligned}$$

Using the decomposition of the objective stress rate (7) in an elastic and a non-elastic part yields

11 $$\begin{aligned} \mathring{{\mathbf {T}}}= \left\{ \begin{array}{*{20}l} m_R \mathring{{\mathbf {T}}}_{\rm el}&{} \quad \text {for } F_H < 0\\ m \big (\mathring{{\mathbf {T}}}_{\rm el}+ y_h ({\mathbf {N}}\mathbin {:}{\mathbf {D}}^0) \, \mathring{{\mathbf {T}}}_{\rm ne}) &{} \quad \text {for } F_H = 0 . \end{array} \right. \end{aligned}$$

This formulation is now ready to be used with barodesy. Barodesy does not allow for a computation of ${\boldsymbol {\mathcal {L}}}^{\rm Baro}$ and ${\mathbf {N}}^{\rm Baro}$. However, it is easy to compute the incrementally elastic response, which delivers equal stress rates for reversal stretchings

12 $$\begin{aligned} \mathring{{\mathbf {T}}}_{\rm el}({\mathbf {D}}) = - \mathring{{\mathbf {T}}}_{\rm el}(-{\mathbf {D}}) \end{aligned}$$

and is therefore the odd part of the constitutive model

13 $$\begin{aligned} \mathring{{\mathbf {T}}}_{\rm el}= \frac{1}{2} \Big ( \mathring{{\mathbf {T}}}({\mathbf {D}}) - \mathring{{\mathbf {T}}}(-{\mathbf {D}}) \Big ). \end{aligned}$$

The nonlinear stress rate is then the rest

14 $$\begin{aligned} \mathring{{\mathbf {T}}}_{\rm ne}= \mathring{{\mathbf {T}}}- \mathring{{\mathbf {T}}}_{\rm el}= \frac{1}{2} \Big ( \mathring{{\mathbf {T}}}({\mathbf {D}}) + \mathring{{\mathbf {T}}}(-{\mathbf {D}}) \Big ), \end{aligned}$$

which is the even part of the constitutive relation. This approach is called internal elastic model in [1], where also an alternative and a bit simpler approach by employing a hypoelastic model is proposed, which is used for the computations in this article. This so-called external elastic model approach uses the incrementally elastic stress rate

15 $$\begin{aligned} \mathring{{\mathbf {T}}}_{\rm el}= {\boldsymbol {\mathcal {M}}}_{\rm el} \mathbin {:}{\mathbf {D}}. \end{aligned}$$

The parameters of the pressure depending elastic stiffness

16 $$\begin{aligned} {\boldsymbol {\mathcal {M}}}_{\rm el} = 2 G \left( {\boldsymbol {\mathcal {I}}} + \frac{\nu }{1-2\nu }{\mathbf {I}}\otimes {\mathbf {I}}\right) \end{aligned}$$

must be calibrated such that the hypoelastic model is consistent with barodesy, see [1] and “Appendix C”. The nonlinear stress rate is the difference of the stress response of barodesy $\mathring{{\mathbf {T}}}_m$ and the elastic stress rate

17 $$\begin{aligned} \mathring{{\mathbf {T}}}_{\rm ne}= \mathring{{\mathbf {T}}}_m - \mathring{{\mathbf {T}}}_{\rm el}. \end{aligned}$$

For certain conditions, the formulation of the model simplifies to certain forms, which are summarized in Table 2.

**Table 2:** Simplification of the general constitutive model to some particular forms

Name	Assumption	Simplified equation	Remarks
Hypoelasticity	$R\rightarrow \infty$	$\mathring{{\mathbf {T}}}=m_R \mathring{{\mathbf {T}}}_{\rm el}$	negligible accumulation
Barodesy	$R\rightarrow 0$	$\mathring{{\mathbf {T}}}=\mathring{{\mathbf {T}}}_{\rm el}+ ({\mathbf {N}}\mathbin {:}{\mathbf {D}}^0) \, \mathring{{\mathbf {T}}}_{\rm ne}$	**monotonic** loading
Barodesy + ISA		$\mathring{{\mathbf {T}}}=m \big (\mathring{{\mathbf {T}}}_{\rm el}+ y_h ({\mathbf {N}}\mathbin {:}{\mathbf {D}}^0) \, \mathring{{\mathbf {T}}}_{\rm ne})$	**monotonic** + **cyclic** loading

## Analysis of the qualitative behaviour of the models

The effect of the different mathematical formulations of ISA and IGS is investigated in this section and reveals some differences and similarities between the predictions of the models. The element tests for these investigations have been carried out using the incremental driver by Niemunis [25] available on www.SoilModels.com [13].

*Calibration*

A default set of clay parameters according to Tables 3 and 4 is used. For the investigations, $\chi$ was set equal to $\chi _0=\chi _{\rm max}$ and for IGS^1^$m_R$ was set to $m_T$, in order to achieve results as comparable as possible.^2^

**Table 3:** Parameters for barodesy, default parameter set for clay [19]

$\varphi _c$	*N*	$\lambda ^*$	$\kappa ^*$
$25^\circ$	1	0.1	0.01

**Table 4:** Parameters for the qualitative analyses with IGS and ISA, Kaolin clay

$m_R=m_T$	*R*	$\beta$	$\chi _0=\chi =\chi _{\rm max}$	$C_a$ (for ISA)
2.6188	$10^{-4}$	0.6	1 or 20	0.018

### Tests with a low number of strain path reversals

#### Undrained triaxial tests (CU)

**Fig. 2:**
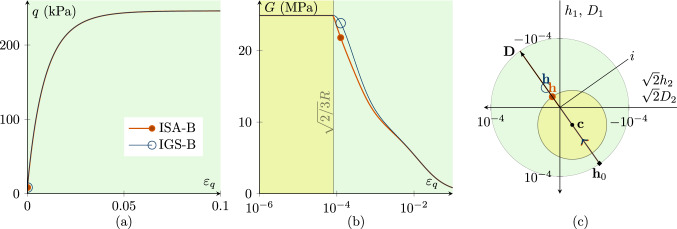
CU test with $${\mathbf {h}}_0=\sqrt{2/3}R \begin{pmatrix} 1 &{} 0 &{} 0 \\ 0 &{} -1/2 &{} 0 \\ 0 &{} 0 &{} -1/2 \\ \end{pmatrix} \,\text {and}\, {\mathbf {c}}_0={\mathbf {h}}_0/2$$ with fully mobilized intergranular strain $||{\mathbf {h}}||=R$, performing a $180^\circ$ strain path reversal; $\chi _0=\chi =\chi _{\rm max}$ is set to 1; $p'_0=200$ kPa; $\text{ OCR}_0=2.5$. **a** Deviatoric stress *q*-deviatoric strain $\varepsilon _q$ plot, **b** the secant shear stiffness *G*–deviatoric strain $\varepsilon _q$ plot, **c** Rendulic planes of intergranular strain ${\mathbf {h}}$ and stretching ${\mathbf {D}}$ for the state indicated by circles in (**a, b**)

**Fig. 3:**
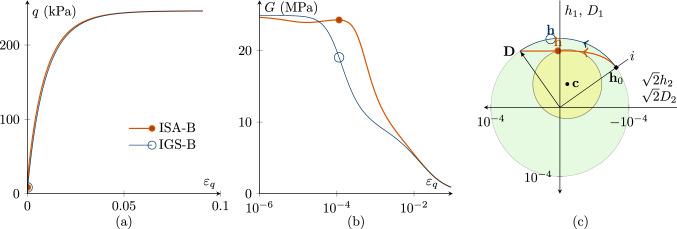
CU test with ${\mathbf {h}}_0=-1/\sqrt{3}R\varvec{I}$ and ${\mathbf {c}}_0={\mathbf {h}}_0/2$ with fully mobilized intergranular strain $||{\mathbf {h}}||=R$, performing a $90^\circ$ strain path reversal; $\chi _0=\chi =\chi _{\rm max}$ is set to 20. $p'_0=200$ kPa, $\text{ OCR}_0=2.5$. The simulations with IGS-B and ISA-B are similar. **a** Deviatoric stress *q*–deviatoric strain $\varepsilon _q$ plot, **b** the secant shear stiffness *G*–deviatoric strain $\varepsilon _q$ plot, **c** Rendulic planes of intergranular strain ${\mathbf {h}}$ and stretching ${\mathbf {D}}$ for the state indicated by circles in (**a, b**)

**Fig. 4:**
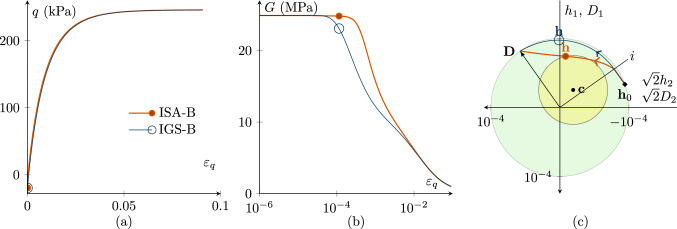
CU test with $${\mathbf {h}}_0=-R \begin{pmatrix} 1/3 &{} 0 &{} 0 \\ 0 &{} 2/3 &{} 0 \\ 0 &{} 0 &{} 2/3 \\ \end{pmatrix}\, \text {and}\, {\mathbf {c}}_0={\mathbf {h}}_0/2$$ with fully mobilized intergranular strain $||{\mathbf {h}}||=R$, performing a $106^\circ$ strain path reversal; $\chi _0=\chi =\chi _{\rm max}$ is set to 20; $p'_0=200$ kPa; $\text{ OCR}_0=2.5$. The simulations with IGS-B and ISA-B are similar. **a** Deviatoric stress *q*–deviatoric strain $\varepsilon _q$ plot, **b** the secant shear stiffness *G*–deviatoric strain $\varepsilon _q$ plot, **c** Rendulic planes of intergranular strain ${\mathbf {h}}$ and stretching ${\mathbf {D}}$ with the initial state ${\mathbf {h}}_0$ marked ◆ (see Supplementary file 2: Animation related to Figure 4)

**Fig. 5:**
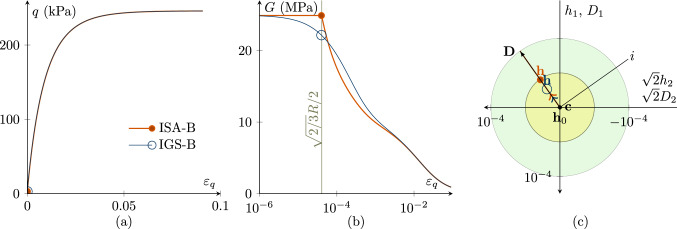
CU test with ${\mathbf {h}}_{0}={\mathbf {c}}_0=\mathbf {0}$; $\chi _0=\chi =\chi_{\rm max}$ is set to 1; $p'_0=200$ kPa; $\text{ OCR}_0=2.5$. Simulations with IGS-B and ISA-B. **a** Deviatoric stress *q*–deviatoric strain $\varepsilon _q$ plot, **b** the secant shear stiffness *G*–deviatoric strain $\varepsilon _q$ plot, **c** Rendulic planes of intergranular strain ${\mathbf {h}}$ and stretching ${\mathbf {D}}$ for the state indicated by circles in (**a, b**). ISA behaves purely elastic until $\varepsilon _q=\sqrt{2/3}\cdot R/2$ (see Supplementary file 3: Animation related to Figure 5)

**Fig. 6:**
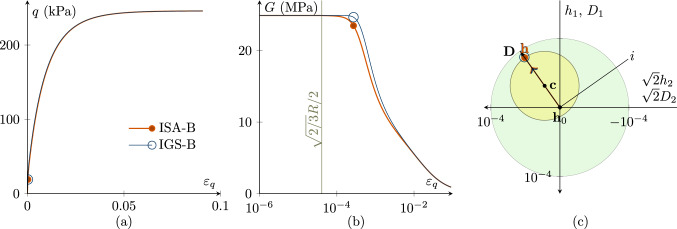
CU test with ${\mathbf {h}}_{0}={\mathbf {c}}_0=\mathbf {0}$; $\chi _0=\chi =\chi_{\rm max}$ is set to 20. $p'_0=200$ kPa, $\text{ OCR}_0=2.5$. Simulations with IGS-B and ISA-B. **a** Deviatoric stress *q*–deviatoric strain $\varepsilon _q$ plot, **b** the secant shear stiffness *G*–deviatoric strain $\varepsilon _q$ plot, **c** Rendulic planes of intergranular strain ${\mathbf {h}}$ and stretching ${\mathbf {D}}$. ISA is described through elasticity until $\varepsilon _q=\sqrt{2/3}\cdot R/2$. Note that the shear stiffness of IGS-B is higher than that of ISA-B

Figures 1, 2, 3, 4, 5 and 6 present the results of investigations of conventional undrained triaxial (CU) tests with different loading histories. The figures contain deviatoric stress *q*-deviatoric strain $\varepsilon _q$ plots, secant shear stiffness *G*-deviatoric strain $\varepsilon _q$ plots and Rendulic planes of intergranular strain ${\mathbf {h}}$ and stretching ${\mathbf {D}}$ spaces.

$180^\circ$ *strain path reversal*

A $180^\circ$ strain path reversal implies isochoric triaxial reloading subsequent to isochoric triaxial unloading. Figure 1 shows the simulation of a CU test, where a $180^\circ$ strain path reversal is performed at fully mobilized intergranular strain $||{\mathbf {h}}||=R$. Thus, the intergranular strain tensor is initialized with $${\mathbf {h}}_0=\sqrt{2/3}R \begin{pmatrix} 1 &{} 0 &{} 0 \\ 0 &{} -1/2 &{} 0 \\ 0 &{} 0 &{} -1/2 \\ \end{pmatrix}$$and for ISA the kinematic hardening tensor is initialized with ${\mathbf {c}}_0={\mathbf {h}}_0/2$. The initial stress state is $p_0'\cdot {\mathbf {I}}=200\cdot {\mathbf {I}}$ kPa with an initial overconsolidation ratio^3^ (OCR) of 2.5. The stress–strain behaviour of IGS and ISA virtually coincides, see Fig. 1a. The shear stiffness follows from the elastic model and is constant until $\varepsilon _q=\sqrt{2/3}\,R$ (yellow shaded area in b). The elastic response, whose stiffness is governed by $m_R$, is followed by a transition between the elastic model and barodesy. Figure 1c shows the Rendulic plane of the intergranular strain ${\mathbf {h}}$ space and the stretching space ${\mathbf {D}}$. The shear stiffness is constant as long as $||{\mathbf {h}}||<R$, followed by an interpolation between elasticity and barodesy. When the bounding surface (larger bubble) is reached and the direction of the stretching coincides with the direction of the intergranular strain, the material behaviour is purely barodetic.

With lower values of $\chi =\chi _0=\chi _{\rm max}$, the stiffness degradation of IGS and ISA slightly differs. For the simulations in Fig. 2, $\chi _0=\chi =\chi _{\rm max}$ is set to 1 for IGS and ISA. The higher $\chi =\chi _0=\chi _{\rm max}$ is, the closer is the agreement between IGS-B and ISA-B.

Note that also $\beta$ influences the stiffness decay with ongoing shear strain. The larger $\beta$ is, the higher is the decay with ongoing shear strain.

A $90^\circ$ strain path reversal occurs, if undrained triaxial compression is performed after isotropic compression. Figure 3 shows the simulation of a CU test, following a strain path reversal of $90^\circ$, starting at fully mobilized intergranular strain with ${\mathbf {h}}_0=-1/\sqrt{3}R\varvec{I}$ and ${\mathbf {c}}_0={\mathbf {h}}_0/2$. The initial stress state is $p'\cdot {\mathbf {I}}=200\cdot {\mathbf {I}}$ kPa again with an initial OCR of 2.5.

The stress–strain response of IGS-B and ISA-B is similar, see Fig. 3a, with small differences, better visible in Fig. 3b and c. For IGS: If the angle between ${\mathbf {h}}$ and ${\mathbf {D}}$ is between $0^\circ$ and $90^\circ$, ${\mathbf {h}}$ stays fully mobilized ($||{\mathbf {h}}||=R$) and rotates towards the direction of ${\mathbf {D}}$ [20, 26], see Fig. 4c.

For ISA-B: The intergranular strain ${\mathbf {h}}$ moves inside the bounding surface on the yield surface towards the bounding surface. As for IGS-B, the stiffness is obtained through interpolation between the elastic model and barodesy if the angle between ${\mathbf {h}}$ and ${\mathbf {D}}$ is between $0^\circ$ and $90^\circ$.

$>90^\circ$ *strain path reversal*

For rotations of the strain path larger than $90^\circ$, the behaviour follows from the elastic model until the angle between ${\mathbf {h}}$ and ${\mathbf {D}}$ is smaller than $90^\circ$, see Fig. 4.^4^ Note that different to the $180^\circ$ strain path reversal, the interpolation between elasticity and barodesy starts before $\varepsilon _q=\sqrt{2/3}R$.

*Initial state* $${\mathbf {h}}_0={\mathbf {c}}_0=\varvec{0}$$

 Figure 5 shows the response of an undrained triaxial test, with ${\mathbf {h}}_{0}=\mathbf {0}$ and in addition for ISA-B ${\mathbf {c}}_0=\mathbf {0}$. In order to highlight the differences between the models, $\chi$ is set to 1. ISA-B is initially elastic until ${\mathbf {h}}$ reaches the yield surface ($||{\mathbf {h}}||=R/2$) and thus at $\varepsilon _q=\sqrt{2/3}\cdot R/2$, see Fig. 5b, c. The deviatoric strain at which ISA starts the interpolation/transition between elasticity and barodesy is marked. The stiffness computed by the IGS decreases from the beginning ($\varepsilon _q=0$, ${\mathbf {h}}=\mathbf {0}$) due to the interpolation between barodesy and elasticity until $||{\mathbf {h}}||=R$.

Figure 6 shows again the response of an undrained triaxial test again with ${\mathbf {h}}_{0}=\mathbf {0}$ and for ISA-B ${\mathbf {c}}_{0}=\mathbf {0}$. However, for these simulations, $\chi _0=\chi =\chi_{\rm max}$ is set to 20. Again ISA-B behaves elastic until $\varepsilon_q=\sqrt{2/3}\cdot R/2$. On the contrary, the stiffness computed by IGS results from the interpolation between barodesy and elasticity until $||{\mathbf {h}}||=R$. For high values of $\chi _0=\chi =\chi_{\rm max}$, the decrease in stiffness for small strain is also very low, so there is hardly any difference between ISA-B and IGS-B.^5^

#### Oedometric compression

**Fig. 7:**
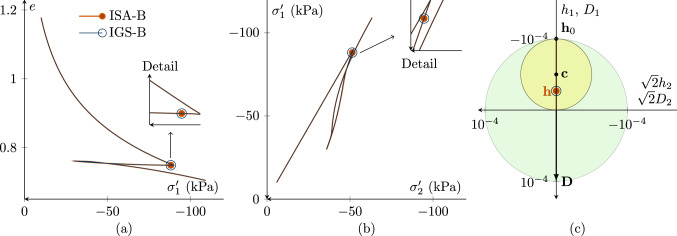
Simulation of loading, un- and reloading of an initially normally consolidated oedometric compression test with the initial values $${\mathbf {h}}_0 = \begin{pmatrix} -R &{} 0 &{} 0 \\ 0 &{} 0 &{} 0 \\ 0 &{} 0 &{} 0 \\ \end{pmatrix}\, \text {and}\, {\mathbf {c}}_0={\mathbf {h}}_0/2$$. The predictions of IGS and ISA overlap. The marked point denotes the state just after oedometric unloading. $\chi_0=\chi =\chi_{\rm max}$ is set to 20

As for the undrained triaxial compression tests, the response of IGS-B and ISA-B is similar for oedometric compression. Figure 7 includes an oedometric simulation with loading, un- and reloading of an initial normally consolidated sample with the parameters according to Tables 3 and 4. The sample is oedometrically normally consolidated and thus $${\mathbf {h}}_0= \begin{pmatrix} -R &{} 0 &{} 0 \\ 0 &{} 0 &{} 0 \\ 0 &{} 0 &{} 0 \\ \end{pmatrix}$$ and ${\mathbf {c}}_0={\mathbf {h}}_0/2$. The predictions of barodesy with IGS and ISA overlap.

Summarizing, it can be seen that with a certain parameter set, the response of ISA and IGS is very similar for low cycles or only one strain paths reversal.

### Cyclic element tests

It is therefore interesting to further investigate conceptional differences between IGS-B and ISA-B with a focus on a higher number of cycles. Different to IGS, ISA includes a yield surface (small bubble) wherein the behaviour is purely elastic for a certain small-strain range. This fact becomes visible in cyclic experiments with small-strain amplitudes $||{\mathbf {h}}||<R$ and is explained in this section.

*CU tests*

**Fig. 8:**
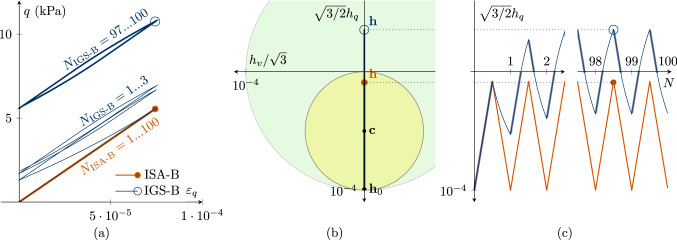
Undrained triaxial compression tests, performing $180^\circ$ strain path reversals with 100 very small-strain cycles $\Delta \varepsilon _q=7.5\cdot 10^{-5}<\sqrt{2/3} R$ with $\chi =\chi_0=\chi_{\rm max}=1$. The initial values are $${\mathbf {h}}_0 = \sqrt{2/3}R \begin{pmatrix} 1 &{} 0 &{} 0 \\ 0 &{} -1/2 &{} 0 \\ 0 &{} 0 &{} -1/2 \\ \end{pmatrix}$$ and thus $h_{0,v}=0$, $h_{0,q}=\sqrt{2/3}R$ **a** deviatoric strain $\varepsilon _q$–deviatoric stress *q* accumulation, **b** $h_v/\sqrt{3}$-$\sqrt{3/2}h_q$ plane of intergranular strain space, **c** deviatoric intergranular strain $\sqrt{3/2}h_q$ evolution with ongoing cycles *N*. For barodesy with IGS, the bold line indicates elastic response, and the thin line indicates transition from elasticity to barodesy. ISA-B behaves purely elastic (see Supplementary file 4: Animation related to Figure 8)

**Fig. 9:**
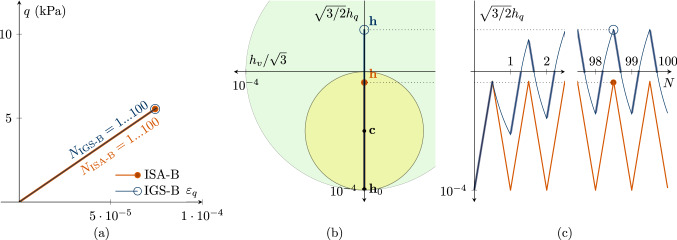
Undrained triaxial compression tests with the initial values $h_{0,v}=0$, $h_{0,q}=\sqrt{2/3}R$ performing 100 very small-strain cycles $\Delta \varepsilon _q=7.5\cdot 10^{-5}<\sqrt{2/3} R$. For high values of $\chi =\chi _0=\chi_{\rm max}=20$, for ISA and for IGS no accumulation of deviatoric stress *q* is obtained. **a** Deviatoric strain $\varepsilon _q$–deviatoric stress *q* plot, **b** $h_v/\sqrt{3}$-$\sqrt{3/2}h_q$ plane of intergranular strain space, **c** deviatoric intergranular strain $\sqrt{3/2}h_q$ development with ongoing cycles *N*

Figures 8 and 9 contain simulations of undrained triaxial compression with 100 applied deviatoric strain cycles $\Delta \varepsilon _q=7.5\cdot 10^{-5}<\sqrt{2/3}\,R$. For a better visibility, only the first three and last three cycles are displayed. The initial values are $${\mathbf {h}}_0 = \sqrt{2/3}R \begin{pmatrix} 1 &{} 0 &{} 0 \\ 0 &{} -1/2 &{} 0 \\ 0 &{} 0 &{} -1/2 \\ \end{pmatrix}$$ and thus the initial volumetric intergranular strain $h_{0,v}=0$ and the deviatoric part $h_{0,q}=\sqrt{2/3}R$.

In order to better show the differences in the response, we start with a small value for $\chi =\chi_0=\chi_{\rm max}=1$ in Fig. 8. As $\Delta \varepsilon_q<\sqrt{2/3}\,R$, ${\mathbf {h}}$ in the simulation with ISA-B stays inside the elastic bubble (Fig. 8b, c). Thus, there is a purely elastic response and no accumulation in deviatoric stress, see Fig. 8a. For IGS-B, the response follows from the elastic model after each loading reversal and is then controlled by transition as soon as ${\mathbf {h}}$ and ${\mathbf {D}}$ point in the same direction (in general for ${\mathbf {h}}:{\mathbf {D}}>0$). In Fig. 8c, the elastic response of IGS ($\circ$) is indicated by the bold line, and the thin line indicates interpolation between elasticity and barodesy, which is responsible for the deviatoric stress *q* accumulation in Fig. 8a.

Note that for high values of $\chi =20$, also for IGS-B no accumulation of deviatoric stress *q* is obtained even though the response of IGS-B is obtained from the elastic model and also from transition between elasticity and barodesy, see Fig. 9.

This is also visible from Fig. 6b, where the response for IGS-B (no elastic behaviour for $0<\Delta \varepsilon _q<\sqrt{2/3}\,R$) is as stiff as for ISA-B (purely elastic for $0\le \Delta \varepsilon _q<\sqrt{2/3}\,R$).

**Fig. 10:**
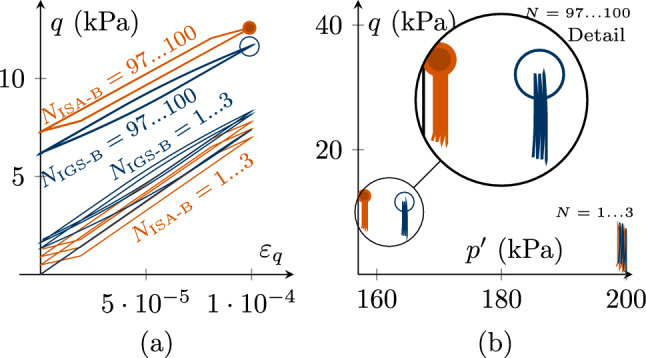
Cyclic CU tests of an initially normally consolidated sample ${\mathbf {h}}_0=-1/\sqrt{3}R\varvec{I}$ and ${\mathbf {c}}_0={\mathbf {h}}_0/2$. Applied strain increments of $\Delta \varepsilon _q=10^{-4}$. $\chi =\chi_0=\chi_{\rm max}=1$. The results for ISA and IGS are comparable. **a** Deviatoric stress *q*–deviatoric strain $\varepsilon _q$, **b** stress accumulation in $p'$-*q* space

**Fig. 11:**
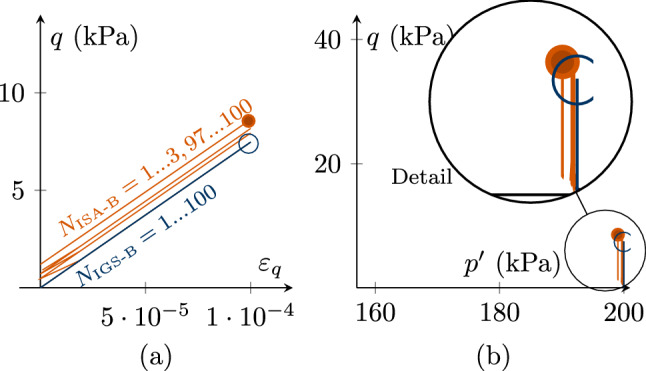
Cyclic CU tests of an initially normally consolidated sample with ${\mathbf {h}}_0=-1/\sqrt{3}R\varvec{I}$ and ${\mathbf {c}}_0={\mathbf {h}}_0/2$. Applied strain increments of $\Delta \varepsilon _q=10^{-4}$. $\chi =\chi _0=\chi_{\rm max}$ is chosen to 20. The results for ISA and IGS are comparable. **a** Deviatoric stress *q*–deviatoric strain $\varepsilon _q$, **b** stress accumulation in $p'$-*q* space

**Fig. 12:**
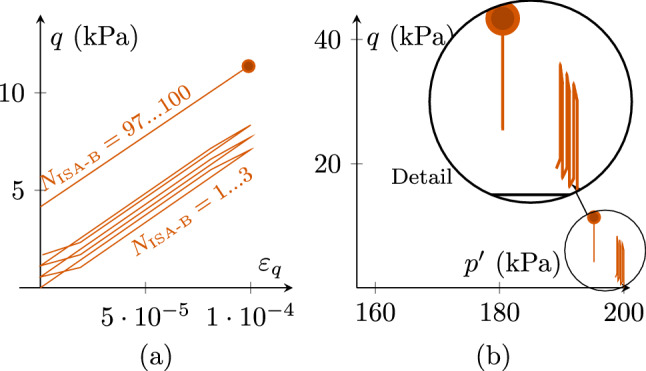
Cyclic CU test of an initially normally consolidated sample ${\mathbf {h}}_0=-1/\sqrt{3}R\varvec{I}$ and ${\mathbf {c}}_0={\mathbf {h}}_0/2$, applying strain increments of $\Delta \varepsilon _q=10^{-4}$, 100 cycles. $\chi _0=1$ and $\chi _{\rm max}=20$. **a** Deviatoric stress *q*–deviatoric strain $\varepsilon _q$, **b** stress accumulation in $p'$-*q* space

In Figs. 10, 11 and 12, cyclic undrained triaxial tests with applied strain increments of $\Delta \varepsilon _q=10^{-4}$ are investigated applying 100 cycles. The sample is initially normally consolidated, i.e. ${\mathbf {h}}_0=-1/\sqrt{3}R\varvec{I}$ and ${\mathbf {c}}_0={\mathbf {h}}_0/2$. The deviatoric strain increment $\Delta \varepsilon _q=10^{-4}$ reaches into the transition zone for both ISA-B and IGS-B. In Fig. 10, $\chi =\chi_0=\chi_{\rm max}$ is set to 1. It is interesting to note that the obtained accumulation in stress is slightly larger for ISA-B. Still, the results are comparable.

For the simulations in Fig. 11, $\chi =\chi _0=\chi_{\rm max}=20$. The obtained accumulation is highly reduced for both models compared with the simulations in Fig. 10. Again, the stress accumulation is slightly higher for ISA-B.

If $\chi$ develops from $\chi _0=1$ to $\chi_{\rm max}=20$, the results for ISA-B according to Fig. 12 are obtained: There is a higher stress accumulation for the first cycles (compared to Fig. 10) and reduced accumulation for a higher number of cycles (compared to Fig. 11). Note that IGS-B used in this work does not allow an evolution of $\chi$. However, very similar results as obtained with ISA-B are expected with IGS according to [5].

**Fig. 13:**
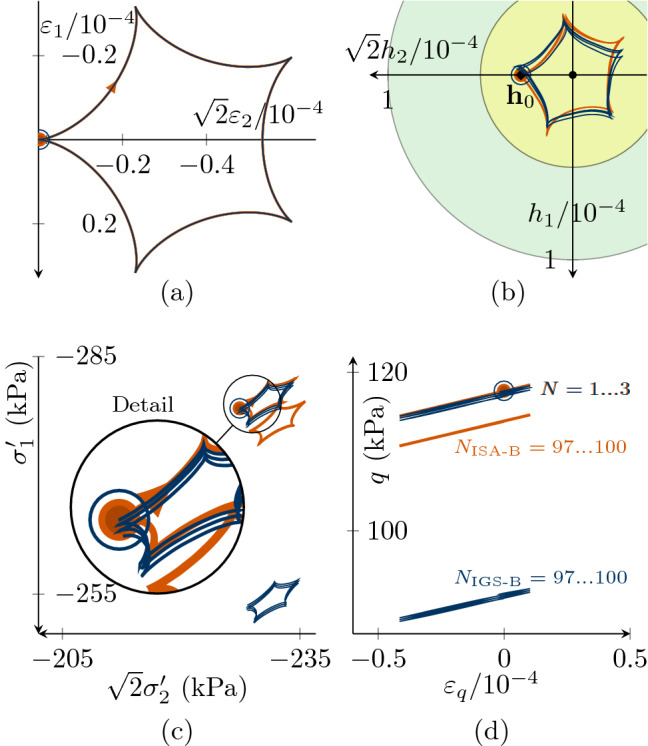
Star-shaped strain paths with 100 very small-strain cycles inside the elastic ISA bubble $||{\mathbf {h}}||<R/2$. The initial values are $${\mathbf {h}}_0=R \begin{pmatrix} 0 &{} 0 &{} 0 \\ 0 &{} 1/5 &{} 0 \\ 0 &{} 0 &{} 1/5 \\ \end{pmatrix}$$ and ${\mathbf {c}}_0=\varvec{0}$. $\chi =\chi _0=\chi _{\rm max}=1$

*Arbitrary paths:* It is of further interest to investigate non-conventional strain paths as the star-shaped strain path displayed in Fig. 13a. We apply these star-shaped strain paths with 100 very small-strain cycles inside the elastic ISA-B bubble $||{\mathbf {h}}||<R$, see Fig. 13b. The initial values are $${\mathbf {h}}_0=R \begin{pmatrix} 0 &{} 0 &{} 0 \\ 0 &{} 1/5 &{} 0 \\ 0 &{} 0 &{} 1/5 \\ \end{pmatrix}$$ and for ISA-B ${\mathbf {c}}_0=\varvec{0}$. In Fig. 13, $\chi$ is set to $\chi _0=\chi_{\rm max}=1$. The obtained stress response is displayed in Fig. 13c. As both ISA and IGS include a hypoelastic formulation, there is a stress accumulation^6^, also for ISA where only the elastic model affects the results. The accumulation is certainly higher for IGS as there is transition between barodesy and hypoelasticity (for ${\mathbf {h}}:{\mathbf {D}}>0$) and $\chi$ is set to a low value of 1.

However, the results between ISA-B and IGS-B become very similar when $\chi =\chi _0=\chi_{\rm max}$ is set to 20, even though IGS-B does not include a purely elastic strain range.

**Fig. 14:**
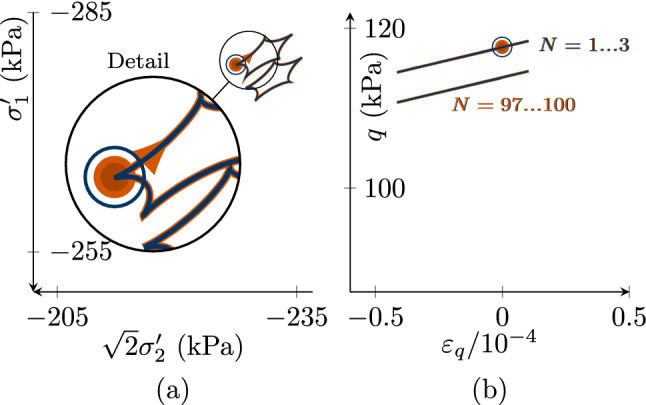
Star-shaped strain paths with 100 very small-strain cycles inside the elastic ISA bubble $||{\mathbf {h}}||<R/2$. $\chi =\chi _0=\chi _{\rm max}=20$. The initial values are $${\mathbf {h}}_0=R \begin{pmatrix} 0 &{} 0 &{} 0 \\ 0 &{} 1/5 &{} 0 \\ 0 &{} 0 &{} 1/5 \\ \end{pmatrix}$$ and ${\mathbf {c}}_0=\varvec{0}$

## Comparison with laboratory data

In the following, both models are validated through simulations of the laboratory data presented by Wichtmann & Triantafyllidis [37] on Kaolin silt. Due to its low plasticity, the behaviour of this material is only marginally time dependent. Consequently, it has no disadvantage to perform the experiments with a time-independent stress–strain rate relation like the new models ISA-B or IGS-B [1].

The experiments comprise an oedometric test with loading, unloading–reloading cycles, undrained monotonic triaxial tests with variation of initial mean pressure $p_0'$ and undrained cyclic triaxial tests with variation of the deviatoric stress amplitude $q^{\rm ampl}$, initial mean stress $p_0'$, initial overconsolidation ratio $\text{ OCR}_0$ and initial stress ratio $\eta _0$. Furthermore, four undrained monotonic triaxial tests with variation of initial mean pressure $p_0'$ performed on Lower Rhine Clay [35] are simulated with the new model.

The adopted parameters for the simulations are listed in Table 5 for the basic barodesy model and in Table 6 for ISA or IGS extension. Until otherwise specified, black dashed lines represent the laboratory data from [37] in each figure. Orange or blue solid lines represent the simulations with either ISA-B or IGS-B, respectively.

For all simulations, the intergranular strain tensor ${\mathbf {h}}$ has been initialized at fully mobilized state ${\mathbf {h}}_0=-1/\sqrt{3}R\varvec{I}$. The kinematic hardening tensor of ISA has been initialized to ${\mathbf {c}}_0=1/(2\sqrt{3})R\varvec{I}$. The internal variable for cyclic history was initialized considering only monotonic previous loading of the sample to $\varepsilon _{a,0}=0$.

**Table 5:** Parameters of barodesy

Material	$\varphi _c$	*N*	$\lambda ^*$	$\kappa ^*$
Kaolin	$26^\circ$	1.14	0.07	0.02
Lower Rhine Clay	$23^\circ$	1.8	0.1	0.02

**Table 6:** Parameters of ISA/IGS for Kaolin

Model	$m_R$	$m_T$	*R*	$\beta$	$\chi _0$	$\chi _{\rm max}$	$C_a$
ISA	2.6	–	$10^{-4}$	0.1	4.3	14	0.018
IGS	3.6	3.6	$10^{-4}$	0.08	10	–	–

### Oedometric test

**Fig. 15:**
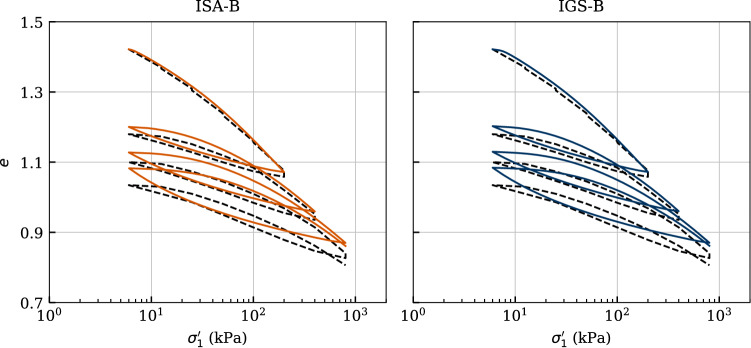
Simulation of an oedometric compression test performed on Kaolin. Parameters calibrated according to Tables 5 and 6

A one-dimensional (oedometric) compression test with three unloading–reloading stress cycles is included in Fig. 15. In both, the experiment and the numerical calculations, all processes were stress-controlled. The three parameters $N,\,\lambda ^*$ and $\kappa ^*$ of the basic barodesy model are calibrated to the values depicted in Table 5 using this experiment.

Both models give comparable results, since the loading is mainly monotonic or occurs with large unloading–reloading loops. This also confirms the qualitative behaviour illustrated in Fig. 7. A very good agreement of the numerical calculations with the laboratory data is evident even at small stresses during loading. The compressibility, hence the change in the void ratio, is very well reproduced. Also, the hysteretic behaviour is described well by the models, yet slightly overestimated. The overestimation arises from the range of small stresses (e.g. $\sigma _1'\le 30$ kPa) during unloading, where both models are too soft compared to the experiment. Hence, the absence of ratcheting at reloading in Figs. 7 and 15, which is present in hypoplastic models with IGS, is partially due to the overestimation of the swelling during unloading.

### Monotonic triaxial tests

**Fig. 16:**
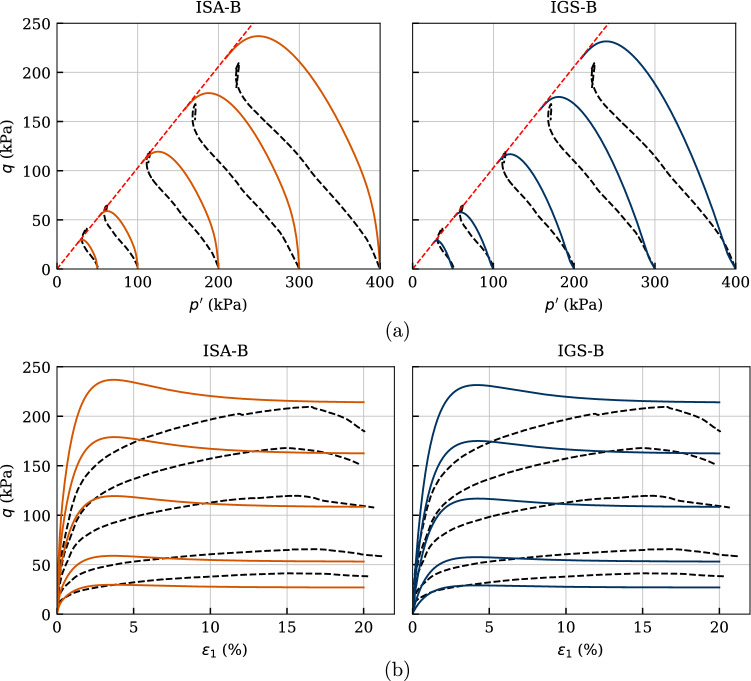
Simulation of monotonic triaxial tests performed on Kaolin with variation of initial mean pressure $p_0'=\{50,\ 100,\ 200,\ 300,\ 400\}$ kPa. Parameters according to Tables 5 and 6

The predictive capabilities of the constitutive model under monotonic behaviour are also checked through the simulations of undrained triaxial tests for different confining pressures. Five undrained triaxial paths of initially normally consolidated and reconstituted samples with $p_0'=\{50,\ 100, 200,\ 300,\ 400\}$ kPa are considered in Fig. 16.

One may check with these simulations the performance of the four material parameters of the basic barodesy model considering that no cyclic loading has been performed. Thus, both ISA-B and IGS-B are expected to provide similar paths for large strains, as is also shown through the qualitative simulations of undrained triaxial tests subsequent to $90^\circ$ strain path reversal in Fig. 3 for $\chi _0=\chi =\chi _{\rm max}=20$. Therefore, the degradation of shear modulus in the transition area occurs faster with IGS. In fact, the effective stress paths, see Fig. 16a, of the two models differ from each other mainly at the beginning. The paths calculated with ISA-B start from the isotropic state vertically upwards (pure shear), whereas those calculated with IGS-B start slightly inclined to the left, which confirms the qualitative behaviour presented in Fig. 3b. In both models, the stiffness and stress response is obtained through interpolation between the elastic model and barodesy, as the angle between ${\mathbf {h}}$ and ${\mathbf {D}}$ is between $0^\circ$ and $90^\circ$. It can also be seen in Fig. 3 that the responses of IGS and ISA differ after a strain path reversal of $90^\circ$ in sense of shear modulus degradation at transition area. Of course, both models provide the same shear strength at critical state. Apart from the previously described slight differences between the models, they also provide comparable results in the strain–stress space, see Fig. 16b.

Comparing these simulations with the experimental results, some issues arise:

(i) the shear strength, see Fig. 16a, b, is well captured by the basic barodesy model.
(ii) the effective stress path of Kaolin is inclined to the upper left due its inherent anisotropy, which is not reproduced by the barodesy model, see Fig. 16a.
(iii) the stress–strain relationship, see Fig. 16b, is in average well reproduced. However, the initial stiffness of both models overestimates the stiffness of the material. The reason for this also stems from the lack of inclusion of the inherent anisotropy into the basic barodesy model, which can be described by the transversal isotropic elasticity [11, 12, 31].

**Fig. 17:**
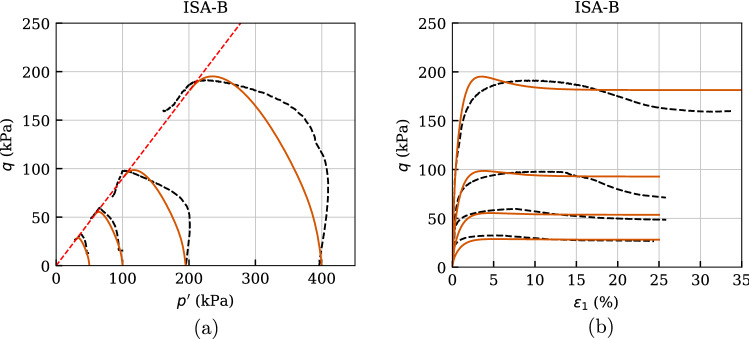
Simulation of monotonic triaxial tests with variation of initial mean pressure $p_0'=\{50,\ 100,\ 200,\ 400\}$ kPa performed on Lower Rhine Clay. Parameters calibrated according to Tables 5 and 6

These issues motivated to simulate similar experiments of another clay and check the performance of the basic barodesy model for fine-grained soils. In that course, Fig. 17 comprises four undrained triaxial tests of initially normally consolidated and reconstituted Lower Rhine Clay samples with $p_0'=\{50,\ 100,\ 200,\ 400\}$ kPa from [35]. Hereby, the numerical calculations are performed only with the barodesy with ISA model as for monotonic loading both models provide similar results. Both the curvature of the effective stress path and the shear strength are reproduced quite satisfactory, see Fig. 17a. This is also reflected in the stress–strain relationship, see Fig. 17b, which is reproduced almost flawlessly by the model. Hence, as denoted also in [35] the Lower Rhine Clay does not possess a cross-isotropic elasticity in contrast to Kaolin.

### Cyclic triaxial test

In several test series in [37], the deviatoric stress amplitude $q^{\rm ampl}$, the initial stress ratio $\eta _0=q_0/p_0'$ as well as the initial overconsolidation ratio $\text{ OCR}_0$ have been varied on reconstituted Kaolin samples. The cyclic loading was applied with a constant displacement rate. In order to test a certain deviatoric stress amplitude, the loading direction was changed once the specified minimum or maximum values of deviatoric stress were reached (pseudo-stress control). The cyclic loading was stopped when a certain value of axial strain (failure criterion, usually $|\varepsilon _1| = 10\%$) was reached.

All simulations have been carried out with both ISA-B model and IGS-B model. The adopted parameters for the basic barodesy model are given in Table 5, and those for the small strain stiffness extensions are listed in Table 6. It is important to note that all simulations have been performed with the same parameter set, i.e. an average best fit of all experiments was targeted.

From the mathematical point of view, it is important to capture the fact that contrary to the qualitative calculations in Figs. 8 and 9, where the cyclic loading was within the yield surface of the intergranular strain, the cyclic loading in this section is in the transition region and passes into the fully mobilized region. Furthermore, the cyclic loading in the qualitative simulations presented in Figs. 10, 11 and 12 reaches into the transition area with $\Delta \varepsilon _q=R$.

***Variation of deviatoric stress amplitude*** $q^{\varvec{ampl}}$ ***at*** $p_0'=200$ ***kPa***

**Fig. 18:**
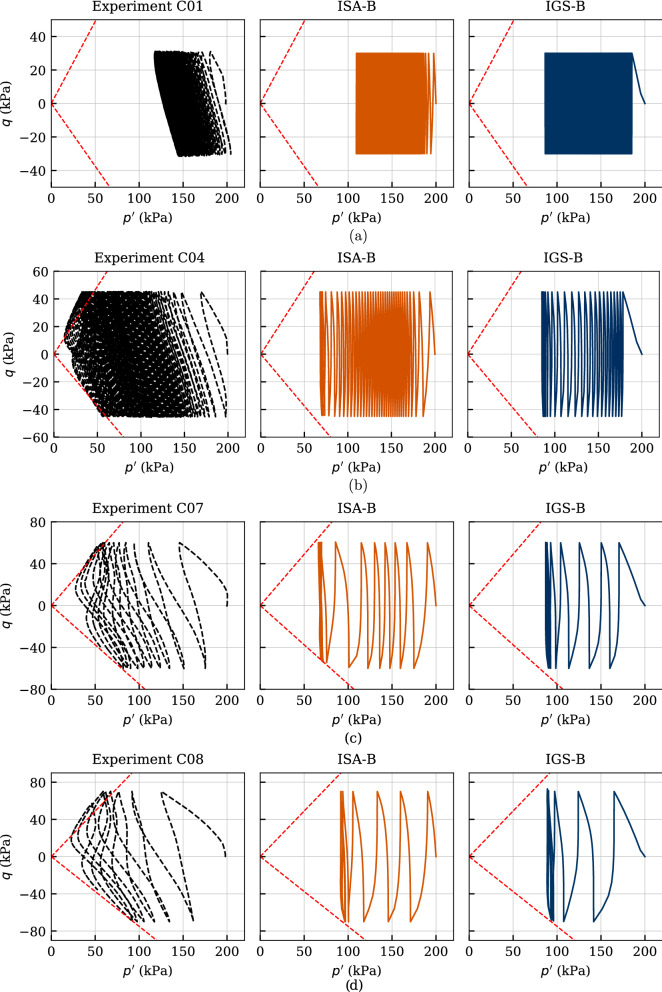
Simulation of cyclic triaxial tests performed on Kaolin with variation of deviatoric amplitude $q^{\rm ampl}=\{30,\ 45,\ 60,\ 70\}$ kPa. Parameters according to Tables 5 and 6

**Fig. 19:**
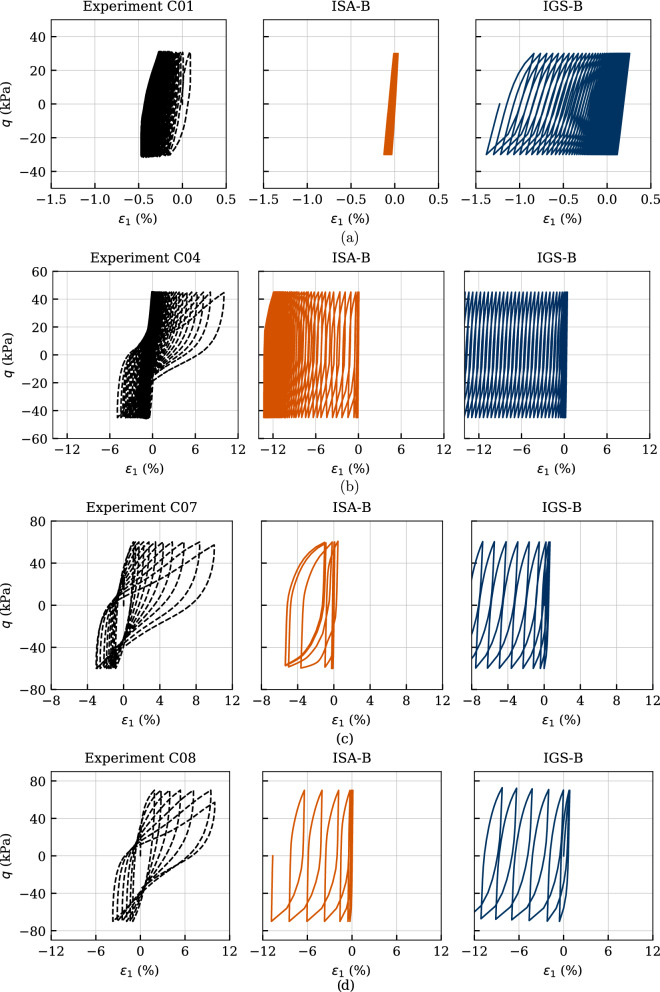
Simulation of cyclic triaxial tests performed on Kaolin with variation of deviatoric amplitude $q^{\rm ampl}=\{30,\ 45,\ 60,\ 70\}$ kPa. Parameters according to Tables 5 and 6

**Fig. 20:**
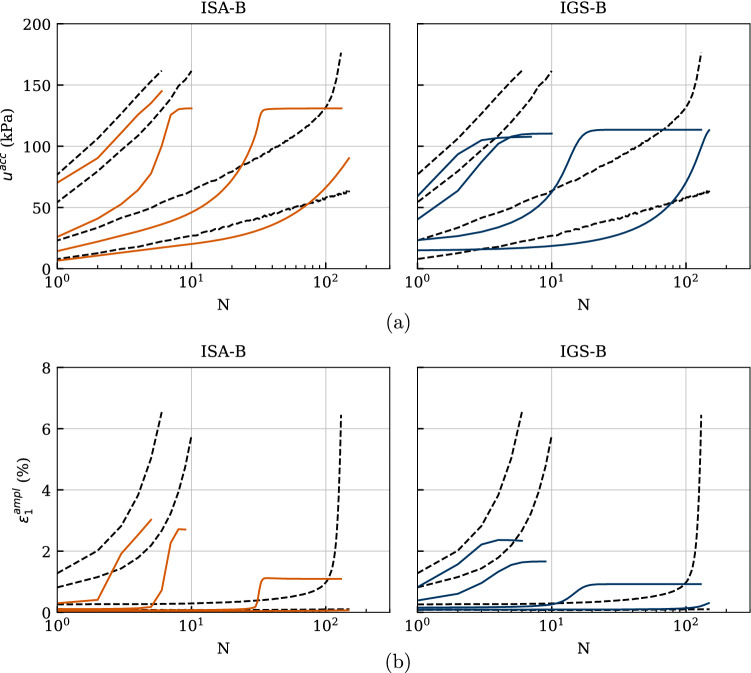
Simulation of cyclic triaxial tests performed on Kaolin with variation of deviatoric amplitude $q^{\rm ampl}=\{30,\ 45,\ 60,\ 70\}$ kPa. Parameters according to Tables 5 and 6

Figures 18, 19 and 20 comprise four experiments named C01, C04, C07 and C08 in [37], with deviatoric stress amplitude between $q^{\rm ampl}=\{30,\ 45,\ 60,\ 70\}$ kPa. The cyclic loading was performed on initially normally consolidated samples of Kaolin with $p_0'=200$ kPa. The calculated number of cycles reached up to $\text{ N }=150$.

In Fig. 18, the effective stress paths of the experiments are compared to the numerical calculations. Except for the slope of the path, which is, as expected, not correctly reproduced, the simulations with both models fit very well to the laboratory data. It can be observed that both models show very well the trend of decreasing number of cycles to failure with increasing deviatoric amplitude, which is also evident in Fig. 20. The larger the loading amplitude, the faster the relaxation of the effective mean pressure and thus the critical state is approached faster. A state with zero effective stress is not reached in these tests on Kaolin with isotropic consolidation. However, the lower the deviatoric amplitude, the closer to the origin of the $p'-q$ plane are the measured final eight-shaped effective stress loops. This behaviour is only partially reproduced by the models—only until the critical state is reached. From this state on, the stress path no longer moves to the left. Note that this behaviour could not be foreseen with the qualitative simulations in Sect. 3 as there the stress state was far away from the critical one.

The stress–strain relationships of these experiments are shown in Fig. 19. Due to the inherent anisotropy, the accumulation of the axial strain occurs in the compression area, where also the failure strain of $\varepsilon _1=10\%$ is reached. The models can only reproduce an accumulation of the axial strain in extension, which is also expected from the qualitative simulations in Figs. 10, 11 and 12. Despite of this, all features of the experiments are very well reproduced with the ISA-B model. The IGS-B model overestimates the strain accumulation at each experiment. For C04, it even reaches axial strains of $\varepsilon _1\approx -100\%$. These shortcomings are not present in the ISA-B model due to the ISA internal variable for cyclic history^7^, which was not expected from the qualitative simulations in Sect. 3 as the respective simulations ranged either in the elastic area or in the transition area and the parameters controlling the accumulation rate where simplified to $\chi =\chi _0=\chi _{\rm max}$. In both Figs. 10 ($\chi =\chi _0=\chi _{\rm max}=1$) and 11 ($\chi =\chi _0=\chi _{\rm max}=20$), the accumulation of ISA-B slightly exceeded the one of IGS-B. Only in the simulation of Fig. 12, where the evolution of the parameter $\chi$ was not turned off ($\chi _0=1,\ \chi _{\rm max}=20$), the accumulation of the ISA-B model was reduced for a higher number of cycles, suggesting the performance of the model observed in this section.

Figure 20b presents the axial strain amplitudes against the number of cycles. Even though ISA-B fits better the experimental behaviour, it also underestimates the axial strain amplitudes for the lower deviatoric stress amplitudes. The trend of faster increasing strain amplitude with increasing stress amplitude is well described by both models.

The curves of accumulated pore water pressure $u^{\rm acc}(N) = u(N)-u(N = 0)$ are provided in Fig. 20a. A value of $u^{\rm acc} = 200$ kPa, which means zero effective stress, has neither been reached in the present tests on Kaolin nor in the numerical calculations. As expected, the accumulation of pore water pressure and thus the relaxation of mean effective stress evolves faster with increasing stress amplitude, which is very well reproduced by the models. Some discrepancies for the last cycles between the simulations with IGS and the experiments for the higher deviatoric stress amplitudes can be observed.

***Variation of initial stress ratio*** $\eta _0$ ***at*** $p_0'=200$ ***kPa***

**Fig. 21:**
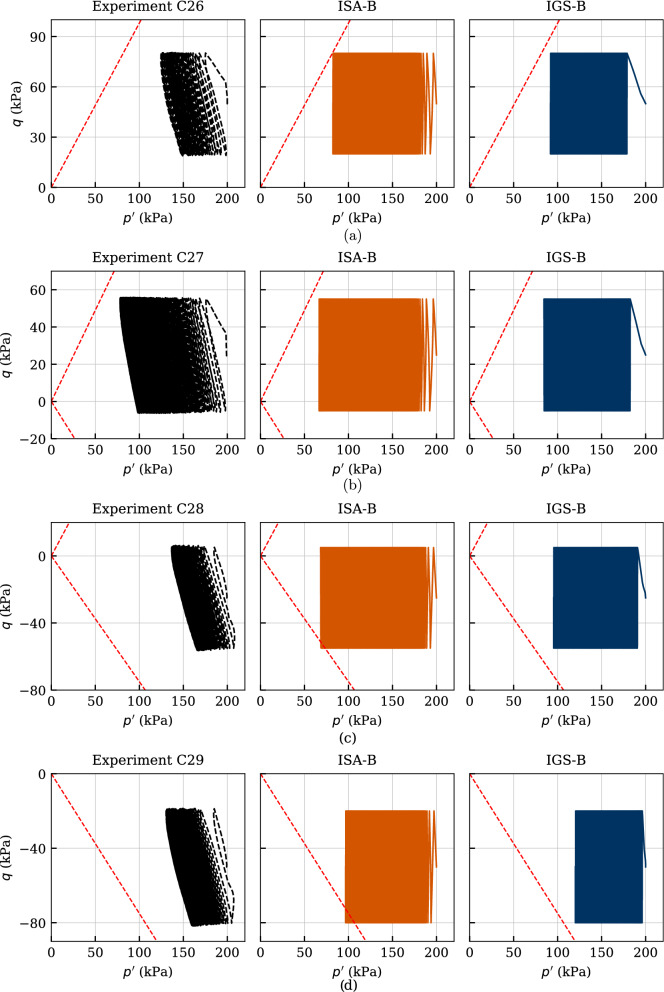
Simulation of cyclic triaxial tests performed on Kaolin with variation of initial stress ratio $\eta _0=\{0.25,\ 0.125,\ -0.125,\ -0.25\}$. Parameters according to Tables 5 and 6

**Fig. 22:**
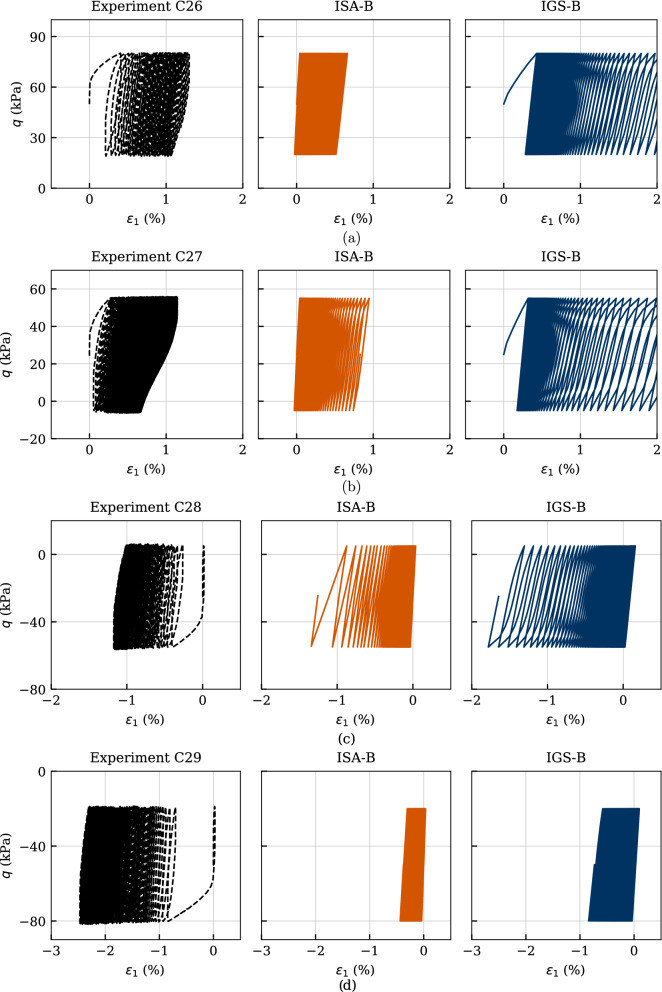
Simulation of cyclic triaxial tests performed on Kaolin with variation of initial stress ratio $\eta _0=\{0.25,\ 0.125,\ -0.125,\ -0.25\}$. Parameters according to Tables 5 and 6

Another four experiments are presented in Figs. 21 and 22. Therefore, the initial stress ratio has been varied between $\eta _0=q/p'=\{0.25,\ 0.125,\ -0.125,\ -0.25\}$ (tests named chronologically C26-C29) for $p_0'=200$ kPa. All samples were initially normally consolidated and have been subjected to a cyclic stress amplitude of $q^{\rm ampl} = 30$ kPa with the displacement rate of $\dot{s} = 0.1$ mm/min. The considered number of cycles for each test amounted to $\text{ N }=150$.

The effective stress paths are depicted in Fig. 21. Similar to the previous observations for the tests with variation of stress amplitude, the simulations with both models are in a very good agreement with the experiments apart from the slope of the path. Again, a state with zero effective mean pressure is not attained either in compression (tests C26 and C27) or in extension (tests C28 and C29) regime, which is well reproduced by the models.

The stress–strain relationships of the experiments and simulations are presented in Fig. 22. Therefore, a considerable accumulation of permanent axial strain was observed, while the axial strain amplitude remained almost constant in contrast to the previous described tests with isotropic consolidation. The accumulation of strain continued even after the pore pressure accumulation had stopped, hence, even though the effective stress path no longer moved to the left. In contrast to the tests with isotropic consolidation, the failure criterion of $|\varepsilon _1| = 10\%$ was reached due to excessive permanent axial strains, and not due to too large strain amplitudes, which is very well reproduced by the models. Furthermore, the accumulation direction is characterized by the direction of the preceding anisotropic consolidation. Hence, the samples consolidated anisotropically in triaxial compression ($\eta _0 > 0$) accumulate permanent axial strains in compression regime and vice versa. This behaviour is also very well simulated by the constitutive models. It is hereby recalled that the isotropically consolidated samples showed a pronounced accumulation of axial strain in compression direction due to the strong inherent anisotropy, see Fig. 19.

Furthermore, especially for the samples with anisotropic consolidation in compression regime, a strong overestimation of the accumulated axial strain can be observed by the simulations with IGS-B. Of course, at the expense of other laboratory tests, one could reproduce these experiments with IGS-B more accurately using a different set of parameters. The parameter set shown in this work has been selected as the mean best fit for all experiments. Nevertheless, the observed excessive strain accumulation presented in Fig. 19 was expected for medium values of the parameter $\chi$ considering the examined qualitative behaviour of IGS-B in Sect. 3, Figs. 13 and 14. Higher values of $\chi$ would render in overall a lower strain accumulation, which may be accurate only for experiment C26 or C27. Hence, a single parameter set which would capture accurately different deviatoric stress amplitudes as well as different initial stress ratios is not possible.

***Variation of initial overconsolidation ratio*** $\text{ OCR}_0$ ***at*** $p_0'=100$ ***kPa and*** $q^{\rm ampl}=30$ ***kPa***

**Fig. 23:**
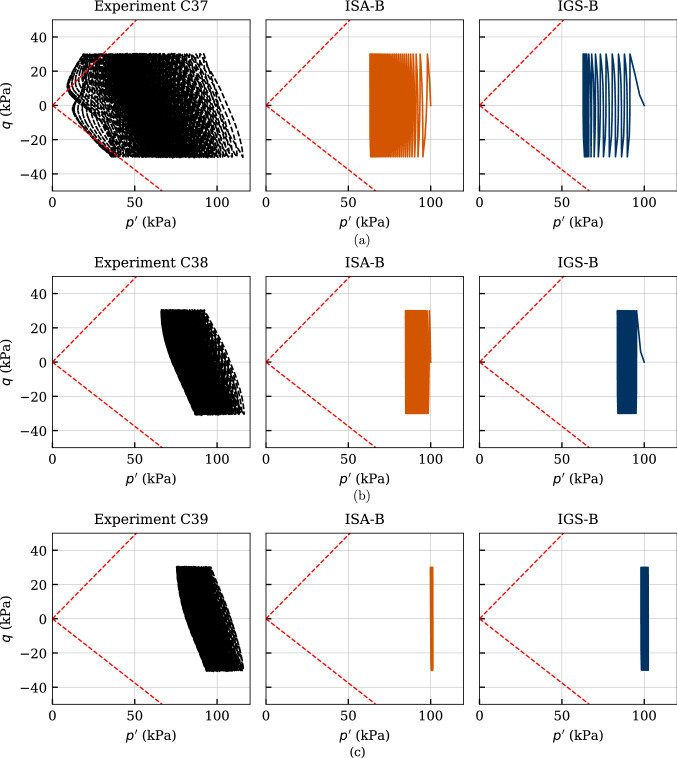
Simulation of cyclic triaxial tests performed on Kaolin with variation of initial overconsolidation ratio $\text{ OCR}_0=\{1.5,\ 2.0,\ 2.5\}$. Parameters according to Tables 5 and 6

**Fig. 24:**
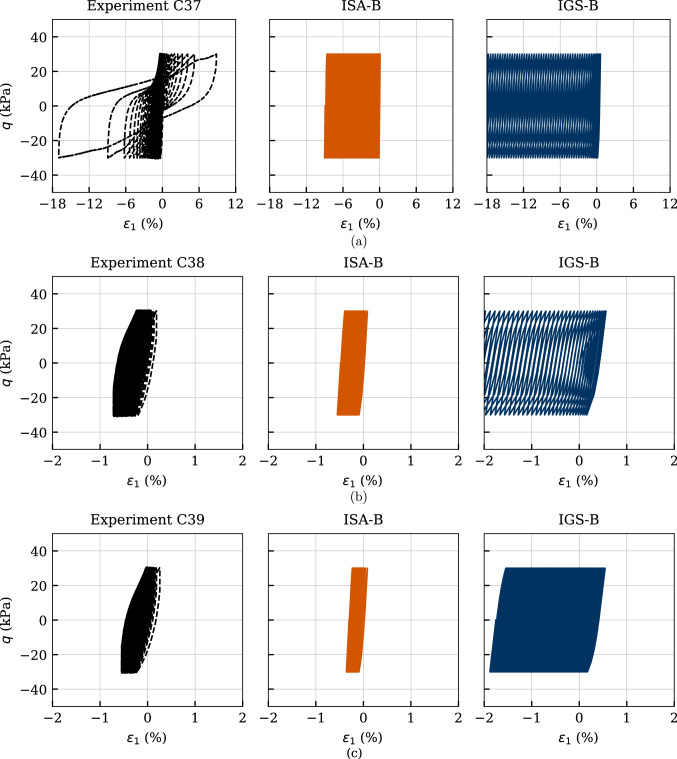
Simulation of cyclic triaxial tests performed on Kaolin with variation of initial overconsolidation ratio $\text{ OCR}_0=\{1.5,\ 2.0,\ 2.5\}$. Parameters according to Tables 5 and 6

**Fig. 25:**
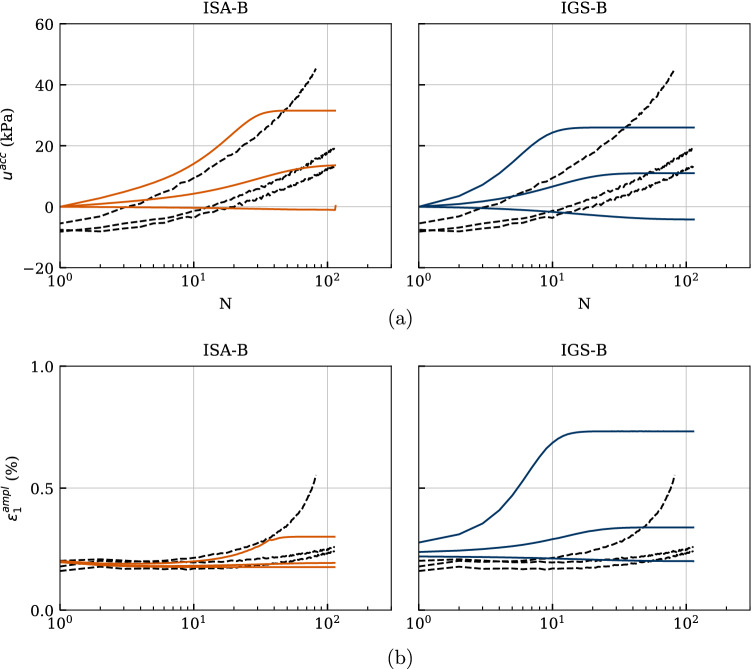
Simulation of cyclic triaxial tests performed on Kaolin with variation of initial overconsolidation ratio $\text{ OCR}_0=\{1.5,\ 2.0,\ 2.5\}$. Parameters according to Tables 5 and 6

The results of three cyclic undrained triaxial tests performed on initially overconsolidated samples $\text{ OCR}_0=\{1.5,\ 2.0,\ 2.5\}$ (tests named chronologically C37-C39 in [37]) are presented in Figs. 23, 24 and 25. The initial mean stress of all tests amounted $p_0'=100$ kPa, and the cyclic loading was performed with $q^{\rm ampl}=30$ kPa and $\dot{s} = 0.1$ mm/min. Again, the number of cycles considered in the numerical calculations was $\text{ N }=150$.

The effective stress paths obtained from these three tests as well as the simulations with the two considered models are provided in Fig. 23. The dilative response observed in the experiment during the first cycle is reproduced by the models only for $\text{OCR}_0=2.5$ (test C39), see also the negative excess pore water pressure in Fig. 25a. The decreasing pore pressure accumulation with increasing $\text{OCR}_0$, resulting in a decay of mean pressure relaxation, is very well captured by the models. Also, the decreasing rate of pore pressure accumulation with increasing $\text{OCR}_0$ is accurately described by the numerical calculations, see Fig. 25a.

Figure 24 presents the stress–strain relationships, whereby the failure criterion of $|\varepsilon _1| = 10\%$ has been reached only for the initially normally consolidated sample (test C37). In all experiments, IGS-B overestimates the excessive permanent axial strains, whereas ISA-B shows a good agreement with the experiments. The accumulation direction is observed in the experiments to occur in extension regime, which is well captured by both models.

The axial strain amplitudes $\varepsilon _1^{ampl}$ versus the number of cycles are illustrated in Fig. 25b. It can be observed that the number of cycles to failure grows considerably with increasing $\text{ OCR}_0$ in both experiments and numerical calculations. Furthermore, IGS-B overestimates the axial strain amplitude especially for $\text{ OCR}_0=1$.

## Summary, conclusion and outlook

In this work, barodesy [22] is extended with ISA plasticity [27] to improve the small-strain predictions of the basic model. Comparisons are made with IGS [26] as well as with experiments [37]. The main differences between ISA and IGS comprise the following aspects:

(i) only ISA includes a purely elastic strain range,
(ii) the ISA version used in this article allows a development from $\chi _0$ to $\chi _{\rm max}$ using the so-called internal variable for cyclic history $0\le \varepsilon _a\le 1$ .

As a result of (i), it is obvious that purely elastic behaviour for very small-strain cycles is only obtained for ISA. However, for high values of $\chi$, the results of ISA and IGS are very similar, even for very small-strain cyclic behaviour. As the elastic formulation is hypoelastic, an accumulation of stress/strain is still obtained for arbitrary stress/strain paths inside the elastic ISA bubble.

The predictions of the experiments performed on Kaolin for a higher number of cycles reveal a few advantages for the ISA model, which results from the fact (ii), hence the inclusion of a transition state from $\chi _0$ to $\chi _{\rm max}$. Equivalent results to ISA are also expected for IGS according to [5]. In fact, if the model allows $\chi _0$ to develop to $\chi _{\rm max}$ and introduces the state variable responsible for cyclic mobility, then also for very small strain or stress cycles, the stress or strain accumulation vanishes even without a predefined purely elastic bubble.

Future work will focus on the extension of the new coupled model for inherent anisotropy, which is shown in the quantitative simulations to present a drawback of the constitutive model. When dealing with medium to highly plastic clays, an influence of the loading frequency on the accumulation behaviour as well as relaxation and creep effects is existent. Therefore, the model will be extended to account for rate- and time-dependent effects of fine-grained soils.

### Supplementary Information

Below is the link to the electronic supplementary material.

Supplementary file 1: Animation related to Figure 1 (GIF 83 KB)

Supplementary file 2: Animation related to Figure 4 (GIF 83 KB)

Supplementary file 3: Animation related to Figure 5 (GIF 83 KB)

Supplementary file 4: Animation related to Figure 8 (GIF 83 KB)

## Symbols and notations

In this article, the symbolic notation is used for the effective Cauchy stress ${\mathbf {T}}$ and stretching ${\mathbf {D}}$, but in some cases the more familiar symbol $\sigma _i'$ instead of $T_i$ is used for the principal stresses. For the principal components of stress, compression is defined negative. Tensors are written in bold capital letters (e.g. ${\mathbf {X}}$). $||{\mathbf {X}}||=\sqrt{\text{tr}\,{\mathbf {X}}^2}$ is the Euclidean norm of ${\mathbf {X}}$, and $\text{tr}\,{\mathbf {X}}$ is the sum of the diagonal components of ${\mathbf {X}}$. The superscript 0 marks a normalised tensor, i.e. ${\mathbf {X}}^0 = {\mathbf {X}}/||{\mathbf {X}}||$. Stresses are considered as effective ones. $\mathring{{\mathbf {T}}}$ is the co-rotational, objective stress rate. The stretching tensor ${\mathbf {D}}$ is the symmetric part of the velocity gradient.

The void ratio *e* is the ratio of the volume of the voids $V_p$ to the volume of the solids $V_s$. Note that with $p'=-\frac{1}{3}\text{tr}\,{\mathbf {T}}$, the mean effective stress is positive for compression. $\varepsilon _{\text {v}}=\text{tr}\,\varvec{\varepsilon }$ is the volumetric strain. For compressive strain, $\varepsilon _i$ is defined negative.

For axisymmetric conditions, often the Rendulic plane is used. For a conventional triaxial compression or oedometric compression test, the axial stress is denoted with $\sigma _1'$ and the radial stress is denoted with $\sigma _2' (=\sigma _3')$. The associated strains are $\varepsilon _1$ and $\varepsilon _2=\varepsilon _3$. Other symbols used are $q=-(\sigma _1-\sigma _2)$ and $\varepsilon _q=-2/3\cdot (\varepsilon _1-\varepsilon _2)$. Initial values are labelled with the subscript 0.

Any symmetric second order tensor can be written as vector with the principal values ${\mathbf {X}}_{\rm v} = \left[ X_1,X_2,X_3\right]$. We use this to display tensors in figures; however, we do not use the notation ${\mathbf {X}}_{\rm v}$, as it is implicitly clear that ${\mathbf {X}}$ is shown as a vector in these figures. Bold calligraphic letters denote tensors of 4th order (e.g. ${\boldsymbol {\mathcal {M}}}$). We use different kinds of tensor operations employing the Einstein summation convention. In particular, the indices follow the lexicographic order: ${\mathbf {X}}\otimes {\mathbf {Y}}= X_{ij}Y_{kl}$, ${\mathbf {X}}\mathbin {:}{\mathbf {Y}}= X_{ij}Y_{ij}$ and ${\boldsymbol {\mathcal {L}}}\mathbin {:}{\mathbf {D}}= {\mathcal {L}}_{ijkl}D_{kl}$. We employ the unit tensor of second order ${\mathbf {I}}$ with $I_{ij} = \delta _{ij}$ and the fourth order tensor ${\boldsymbol {\mathcal {I}}}$ with ${\mathcal {I}}_{ijkl} = \delta _{ik}\delta _{jl}$, using the Kronecker delta $\delta _{ij}$.

## Equations of barodesy

In this appendix, all equations of barodesy for clay [22] are summarized.

18 $$\begin{aligned} {\mathring{{\mathbf {T}}}}= h\cdot (f\mathbf {R}^0+g{\mathbf {T}}^0)\cdot |{\mathbf {D}}| \end{aligned}$$

19 $$\begin{aligned} {\mathbf {R}}=  -\exp (\alpha {\mathbf {D}}^0) \,\, \text{ with }\,\, \alpha =\dfrac{\ln K}{\sqrt{3/2-{\text{tr}\,{\mathbf {D}}^0}^2/2}} \end{aligned}$$

20 $$\begin{aligned} K= 1-\dfrac{1}{1+c_1(m-c_2)^2} \,\, \text{ with }\,\, m=\dfrac{-3\text{tr}\,{\mathbf {D}}^0}{\sqrt{6-2{\text{tr}\,{\mathbf {D}}^0}^2}} \end{aligned}$$

21 $$\begin{aligned} h= c_3|{\mathbf {T}}|^{c_4} \end{aligned}$$

22 $$\begin{aligned} f= c_6\cdot \beta \cdot \text{tr}\,{\mathbf {D}}^0-\frac{1}{2} \end{aligned}$$

23 $$\begin{aligned} g=  (1-c_6)\cdot \beta \cdot \text{tr}\,{\mathbf {D}}^0+\left( \frac{1+e}{1+e_c}\right) ^{c_5}-\frac{1}{2} \end{aligned}$$

24 $$\begin{aligned} e_c= \exp \left( N-\lambda ^*\ln \frac{-2/3 \,\text{tr}\,{\mathbf {T}}}{\sigma ^*}\right) -1\ \end{aligned}$$

25 $$\begin{aligned} \beta= & {} -\frac{1}{c_3\Lambda }+ \frac{1}{\sqrt{3}}2^{c_5\lambda ^*}- \frac{1}{\sqrt{3}} \end{aligned}$$

26 $$\begin{aligned} \Lambda= & {} -\frac{\lambda ^*-\kappa ^*}{2\sqrt{3}}\text{tr}\,{\mathbf {D}}^0+\frac{\lambda ^*+\kappa ^*}{2} \end{aligned}$$

The constants $c_1$ - $c_6$ can be determined on the basis of the critical state soil mechanics parameters $\varphi _c$, *N*, $\lambda ^*$ and $\kappa ^*$ [22].

## Consistent calibration of the hypoelastic model

The parameters *G* and $\nu$ for the hypoelastic model in Eq. (16) can be directly calibrated using barodesy [1]. We use the average, isotropic unloading and reloading stiffness of barodesy at a normally consolidated, isotropic stress state with

27 $$\begin{aligned} K = p'\frac{1}{2}\left( \frac{1}{\kappa ^*}-\frac{2c_3}{\sqrt{3}}\left( 2^{\lambda ^*c_5} -1\right) +\frac{1}{\lambda ^*} \right) . \end{aligned}$$

The incremental shear stiffness of barodesy at an isotropic stress state depends only the pressure $p'$

28 $$\begin{aligned} G=  p' \frac{c_3(K_c -1)}{2\sqrt{2}\sqrt{1+K_c^2}}, \nonumber \\&\text {with } K_c = \frac{1-\sin \varphi _c}{1+\sin \varphi _c}. \end{aligned}$$

Thus, we obtain a pressure-independent Poisson’s ratio

29 $$\begin{aligned} \nu = \frac{3K-2G}{6K+2G}. \end{aligned}$$

With the elastic shear modulus *G* we can calculate the stiffness factor $m_R = \frac{G_0}{G}$, using the pressure-dependent small-strain shear modulus $G_0$ of the soil.

## Calculation of the OCR and void ratio *e* according to a given asymptotic stress state

The Hvorslev overconsolidation according to an given stress ratio $K_{\rm mob}$ can be calculated using the asymptotic state boundary surface of barodesy [3]. This stress ratio is starting with a given axisymmetric triaxial compression state ($\sigma _1'<\sigma _2'=\sigma _3'$) when

$$\begin{aligned} K_{\rm mob}=\dfrac{\sigma _2'}{\sigma _1'} =\dfrac{3-q/p'}{3+2q/p'}. \end{aligned}$$

For axisymmetric triaxial extension ($\sigma _1'>\sigma _2'=\sigma _3'$) the stress ratio is

$$\begin{aligned} K_{\rm mob}=\dfrac{\sigma _1'}{\sigma _2'}. \end{aligned}$$

With

$$\begin{aligned} m = c_2 + \sqrt{\frac{1}{c_1}\left( \frac{1}{1-K_{\rm mob}}-1\right) } \end{aligned}$$

we obtain two solutions for the dilatancy $\text{tr}\,{\mathbf {D}}^0_a$ depending on the stress ratio $K_{\rm mob}$:

$$\begin{aligned} \text{tr}\,{\mathbf {D}}^0_a = \left\{ \begin{array}{ll} \sqrt{\frac{6m^2}{9+2m^2}} \quad &{}\text {for}\; K_{\rm mob} \le K_c \\ -\sqrt{\frac{6m^2}{9+2m^2}} \quad &{}\text {for}\; K_{\rm mob} > K_c \end{array}\right. , \end{aligned}$$

with the critical stress ratio $K_c = \frac{1-\sin \varphi _c}{1+\sin \varphi _c}$. We can calculate $1/OCR_a$ for the asymptotic state related to $\text{tr}\,{\mathbf {D}}^0_a$

30 $$\begin{aligned} \dfrac{1}{OCR_a}=\dfrac{1}{2}\left( \dfrac{-{\text{tr}\,{{{\mathbf {D}}}^0_a}}/{\lambda ^*}+c_3-c_3\text{tr}\,{{{\mathbf {D}}}^0_a}\beta }{c_3}\right) ^\frac{1}{\lambda ^*c_5}, \end{aligned}$$

with

$$\begin{aligned} \beta =-\frac{1}{c_3\Lambda }+ \frac{1}{\sqrt{3}}2^{c_5\lambda ^*}- \frac{1}{\sqrt{3}} \quad \end{aligned}$$

and

$$\begin{aligned} \Lambda =-\frac{\lambda ^*-\kappa ^*}{2\sqrt{3}}\text{tr}\,{\mathbf {D}}^0+\frac{\lambda ^*+\kappa ^*}{2}. \end{aligned}$$

It thus follows the void ratio at an asymptotic state $e_a$ for $OCR_a$ (30)

31 $$\begin{aligned} e_a=\exp {(N-\lambda ^*\ln (OCR_a \cdot p'))}-1. \end{aligned}$$

## Footnotes

1 For a strain path reversal of $180^\circ$, the shear stiffness ${\boldsymbol{\mathcal {M}}}$ is equal to $m_R {\boldsymbol {\mathcal {L}}}$. For a strain path reversal of $90^\circ$, the shear stiffness ${\boldsymbol{\mathcal {M}}}$ is equal to $m_T {\boldsymbol{\mathcal {L}}}$. Note that in general the shear stiffness is lower for a $90^\circ$ than for a $180^\circ$ turn of the strain path, and $m_T$ is usually chosen smaller than $m_R$. For better comparison with ISA, we set $m_T=m_R$ also for IGS.

2 In order to make the characteristics of the models visible, we chose $\chi =\chi _0=\chi _{\rm max}$ either very large with 20 or rather small with 1.

3 The OCR is here defined as $p_e'/p'$ with the Hvorslev equivalent pressure $p_e'=\exp \left( \frac{N-\ln (1+e)}{\lambda ^*}\right)$ as the intersection point of the current specific volume $(1+e)$ and the NCL in $\ln (1+e) - \ln p'$ space.

4 In order to obtain a strain path $\ge 90^\circ$, the following strain path before undrained compression is chosen: $D_1=-1/3$, $D_2=D_3=-2/3$. The initial stress state with $p_0'=200$ kPa is chosen to $T_1=-181$ kPa and $T_2=T_3=-209$ kPa.

5 It is interesting to note that even though IGS-B is not purely elastic in this simulation (just except for the initial state with ${\mathbf {h}}=\mathbf {0}$), it behaves stiffer with ongoing $\varepsilon _q$ than ISA-B. This fact will later help to interpret tests with cyclic loading.

6 With a hyperelastic model, a closed strain loop results in a closed stress loop.

7 Note that with the recent improvement according to [5] these shortcomings are expected to be eliminated also for IGS.
