# Supplementary figures and images for: Comparison of two small-strain concepts: ISA and intergranular strain applied to barodesy
Source: Acta Geotech. 2022 Apr 6;17(10):4333–58. doi: 10.1007/s11440-022-01454-3 (PMC9546824; doi:10.1007/s11440-022-01454-3)

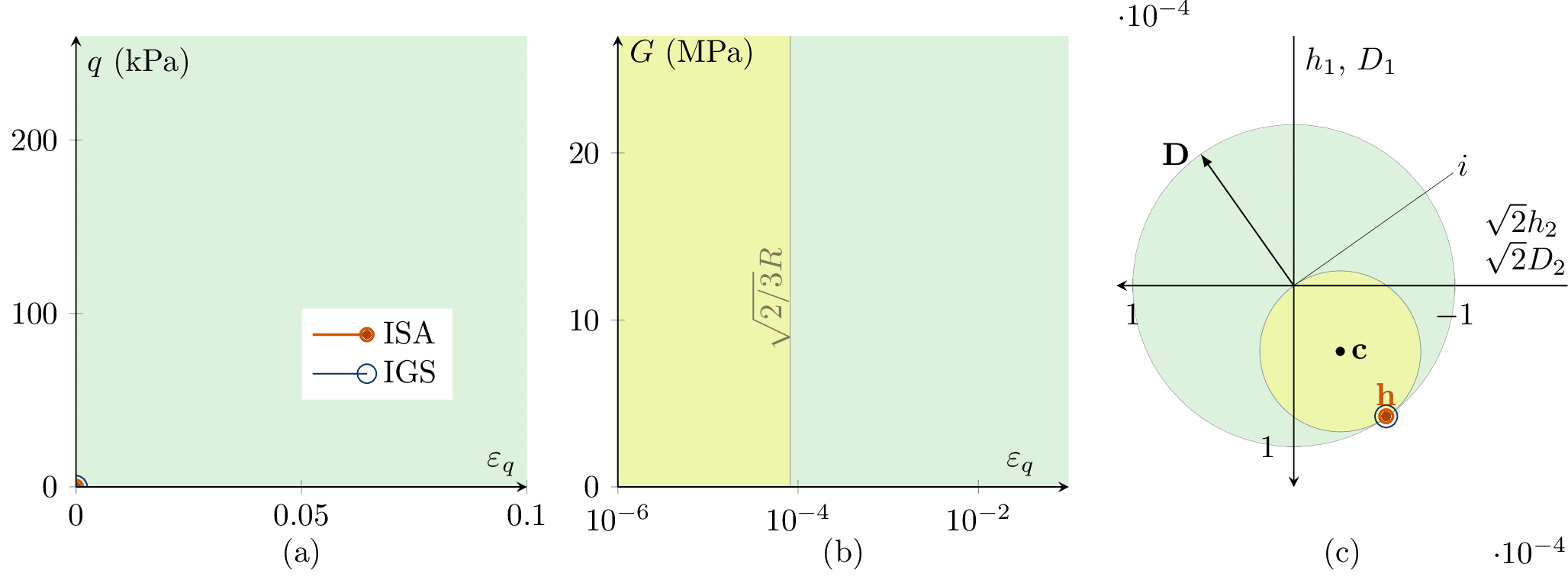

Supplement: Supplementary file 1 — Animation related to Figure 1 (GIF 83 KB) [file 11440_2022_1454_MOESM1_ESM.gif]

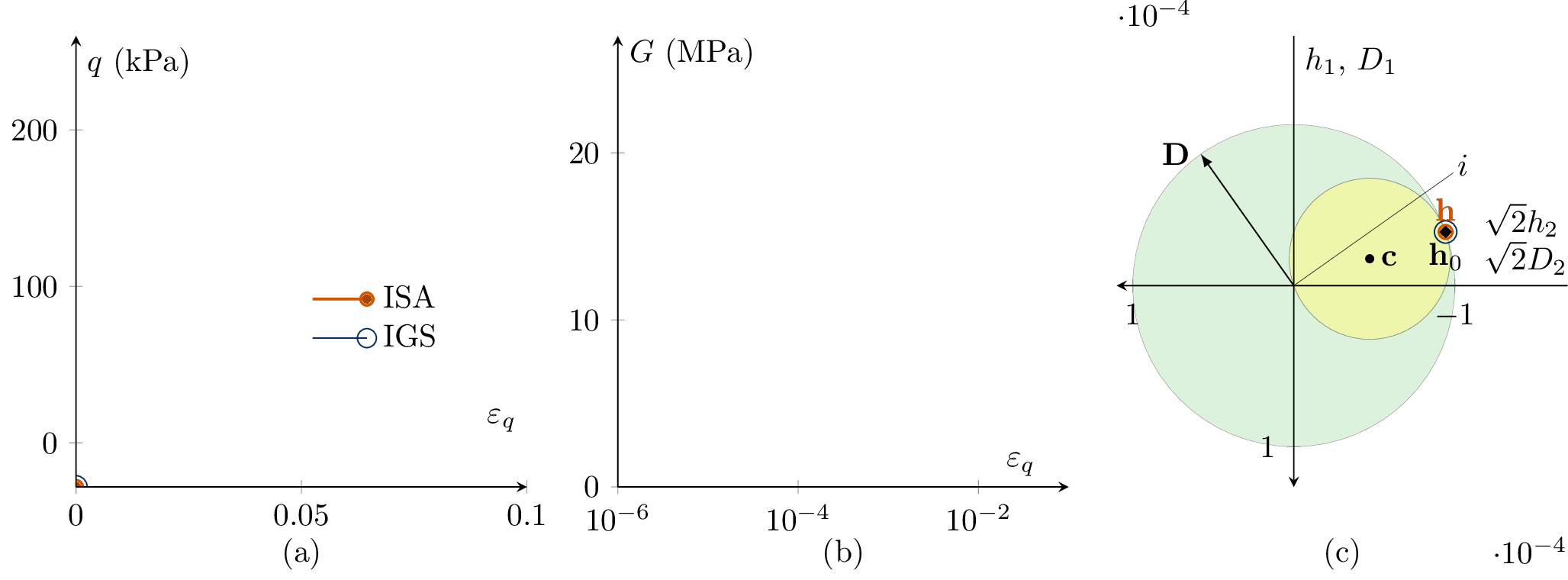

Supplement: Supplementary file 2 — Animation related to Figure 4 (GIF 83 KB) [file 11440_2022_1454_MOESM2_ESM.gif]

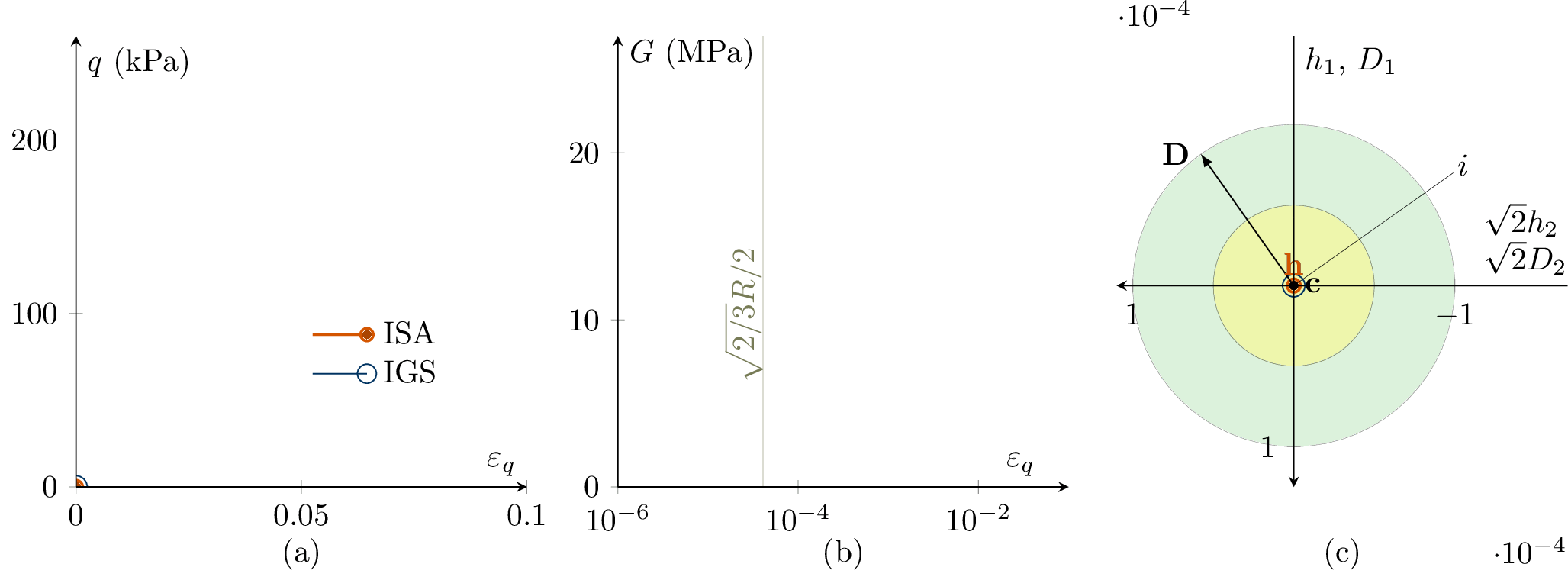

Supplement: Supplementary file 3 — Animation related to Figure 5 (GIF 83 KB) [file 11440_2022_1454_MOESM3_ESM.gif]

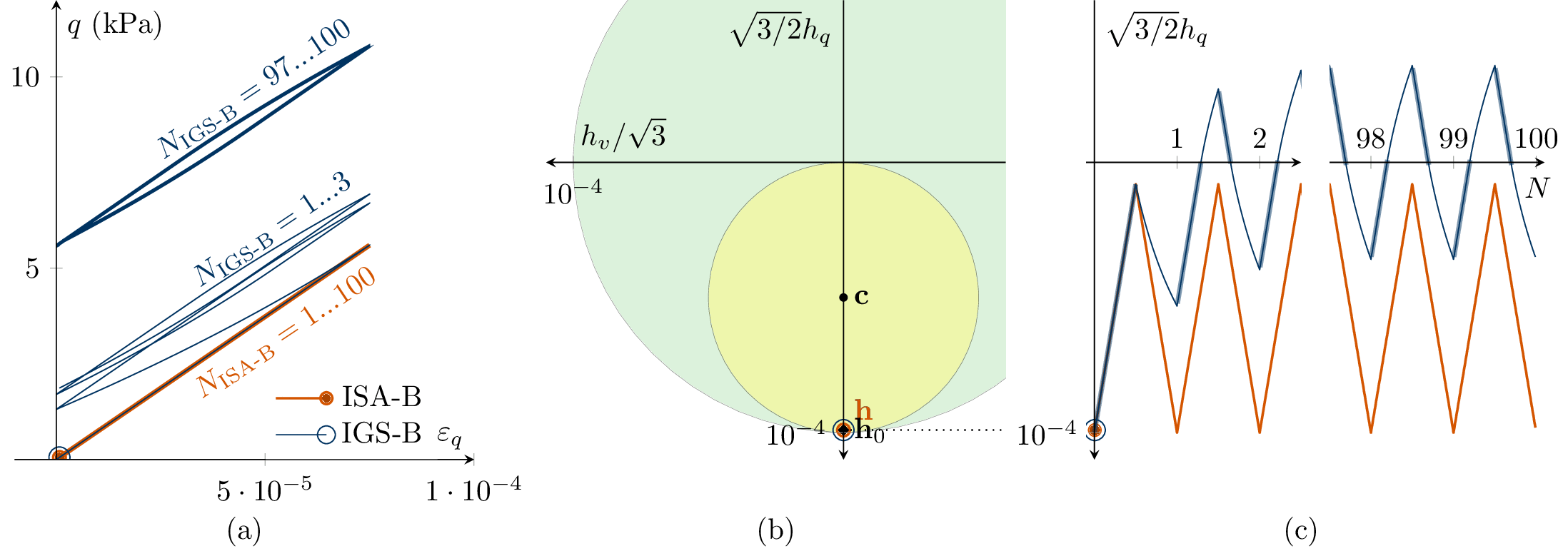

Supplement: Supplementary file 4 — Animation related to Figure 8 (GIF 83 KB) [file 11440_2022_1454_MOESM4_ESM.gif]
